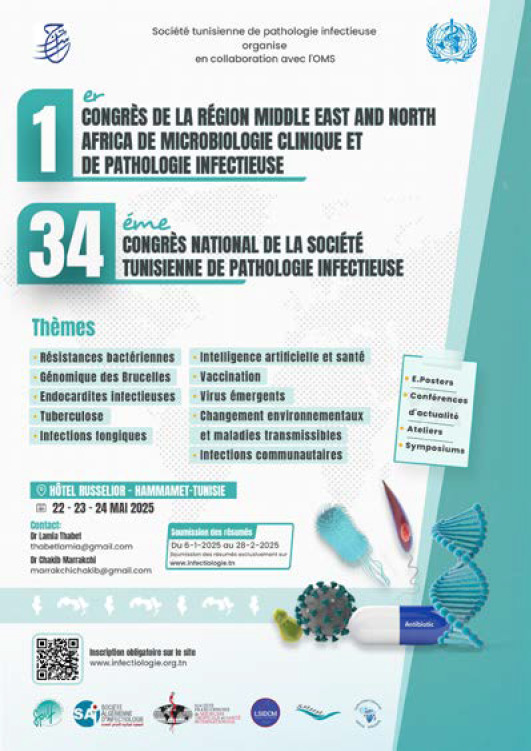# 34^e^ Congrès national de la STPI - 1^er^ Congrès de la région *Middle East and North Africa* de microbiologie clinique et de pathologie infectieuse 22-24 mai 2025, Hammamet, Tunisie: Organisateur: Société Tunisienne de Pathologie Infectieuse (STPI)

**DOI:** 10.48327/mtsi.v5i4.2025.777

**Published:** 2025-11-09

**Authors:** Pr Adnene TOUMI

**Affiliations:** Professeur en maladies infectieuses à la Faculté de médecine de Monastir et au CHU Fattouma Bourguiba Rue 1^er^ juin 1955, Monastir, Tunisie Vice-Président de la STPI

## Éditorial

La Société Tunisienne de Pathologie Infectieuse (STPI) est une société savante scientifique créée en 1988. Elle est active dans les domaines des maladies infectieuses, de la microbiologie et de la parasitologie-mycologie. Elle est engagée dans la santé publique et la recherche dans le domaine de l’infectiologie au sens large. Elle organise chaque année plusieurs manifestations scientifiques, notamment son congrès national annuel dont la 34^e^ édition s’est tenue en 2025.

La STPI a pour principaux objectifs et missions: le développement de la qualité de la prise en charge et de la prévention des maladies infectieuses en Tunisie, la formation des résidents en collaboration avec les collèges et sociétés savantes partenaires, la participation à l’élaboration de consensus et à la réalisation d’études multicentriques, ainsi que la collaboration avec d’autres sociétés savantes, à l’échelle nationale et internationale, notamment par l’organisation de cours de formation et d’évènements conjoints.

Le 34^e^ Congrès national annuel de la STPI, couplé au 1^er^ Congrès de la Région *Middle East and North Africa* en microbiologie clinique et en pathologie infectieuse, s’est tenu du 22 au 24 mai 2025 à Hammamet, en Tunisie. Cet événement scientifique majeur a été co-organisé en partenariat avec l’Organisation mondiale de la Santé, avec la participation active de plusieurs experts nationaux et internationaux, et le soutien de plusieurs sociétés savantes: la Société de Pathologie Infectieuse de Langue Française (SPILF), la Société Francophone de Médecine Tropicale et Santé Internationale (SFMTSI), la Société Française de Microbiologie (SFM), la Société Algérienne d’Infectiologie (SAI), la Société Marocaine de Microbiologie Médicale (SMMM), la Lebanese Society of Infectious Diseases (LSIDSM) et l’Infection Control Africa Network (ICAN).

La manifestation a abordé plusieurs thématiques allant de l’infectiologie clinique à la bactériologie, la virologie, la parasitologie et la mycologie. Le programme a comporté neuf sessions thématiques, trois ateliers pratiques de formation et cinq symposiums. Les thèmes développés ont porté notamment sur l’émergence des maladies vectorielles en Méditerranée, l’apport de l’intelligence artificielle dans le domaine des pathologies infectieuses, la résistance aux antimicrobiens, les infections associées aux soins, la tuberculose, la vaccination et les endocardites infectieuses.

Le congrès a privilégié la multidisciplinarité en insistant sur des aspects liés plus particulièrement à l’épidémiologie, à la clinique, à la biologie, au traitement et au contrôle des maladies infectieuses. La participation a été massive avec plus de 350 participants de Tunisie, d’Algérie, du Maroc et de France.

Trente-quatre conférences ont été animées par d’éminents spécialistes et près de 580 communications scientifiques affichées ont été présentées sous forme d’e-posters dans des sessions dédiées.

Ce congrès s’est distingué par sa capacité à rassembler une communauté scientifique diversifiée autour d’une vision commune: renforcer les liens entre les pays de la région MENA, mutualiser les expériences et construire un réseau régional solide et durable. Il constitue le point de départ d’une dynamique collaborative que nous souhaitons inscrire dans la durée.

Comité scientifique: Adnene Toumi, Aida Berriche, Chakib Marrakchi, Habiba Naïja, Hanene Smaoui, Hayet Sellami, Hela Hannachi, Lamia Ammari, Manel Marzouk, Makram Koubaa, Nadia Ben Lasfar, Naïla Hannachi, Rym Ben Abdallah, Saba Gargouri, Salma Mhalla, Sonia Trabelsi, Sonda Mezghanni, Wafa Marrakchi, Yosr Guedri

**Figure:**