# Supplementary material for: 34th STPI National Congress - 1st Middle East and North Africa Region Congress of clinical microbiology and infectious pathology May 22-24, 2025, Hammamet, Tunisia
Source: Med Trop Sante Int. 2025 Nov 9;5(4):mtsi.v5i4.2025.777. [Article in French] doi: 10.48327/mtsi.v5i4.2025.777 (PMC12948371; doi:10.48327/mtsi.v5i4.2025.777)
Supplement: Related texts

# Research article: Biofire® Filmarray® Pneumonia plus Panel: quels gains par rapport à l’usage d’antibiotiques?

**Published:** 2025-11-09

**Authors:** I. Bergaoui, A. Hmidet, E. Hachena, M. Fourati, A. Amara, H. Njima

**Affiliations:** Laboratoire Bio24-alliance, centre Ruspina médical, Monastir, Tunisie

Les infections respiratoires représentent un enjeu majeur de santé publique en raison de leur fréquence et leur morbi-mortalité élevée. Elles représentent une cause principale de prescription d’antibiotiques. Cependant un bon nombre d’entre elles sont dues à des infections virales pour lesquelles il n’y a pas d’indication d’antibiothérapie.

L’objectif de cette étude était de déterminer l’apport de la PCR multiplex dans le diagnostic des infections respiratoires et dans la lutte contre l’antibiorésistance.

Il s’agit d’une étude descriptive rétrospective faite sur une période de deux ans (janvier 2023-décembre 2024) portant sur des prélèvements respiratoires (aspirations trachéales et prélèvements distaux protégés) prélevés de patients hospitalisés au service de réanimation. La détection des pathogènes respiratoires a été réalisée par une PCR multiplex à l’aide du kit BioFire® FilmArray® Pneumonia plus Panel. Ce kit permet l’identification de 18 bactéries, 9 virus et 7 gènes de résistance aux antibiotiques.

Au total, 65 patients ont été inclus dans cette étude. Environ 70% des demandes ont été reçues en période hivernale. La PCR multiplex était positive chez 46 patients. Une étiologie virale de la symptomatologie respiratoire a pu être documentée chez 23 patients (50%). Un seul virus était détecté chez 18 patients. Il s’agissait de rhino/entérovirus dans 5 cas, coronavirus dans 7 cas, grippe A dans 3 cas, virus para-influenzae dans 2 cas et virus respiratoire syncitial (VRS) dans 1 cas. Une co-infection par 2 virus était observée chez 5 patients: VRS-coronavirus chez 3 patients, 1 cas de co-infection rhino/enterovirus-coronavirus et 1 cas de co-infection rhino/ entérovirus-adénovirus. Sur les 23 patients, 5 présentaient une infection mixte documentée bactérienne et virale. Parmi les co-infections bactériennes, la PCR a détecté la présence du gène mecA chez une souche de *Staphylococcus aureus,* de CTX-M chez 2 isolats de *Escherichia coli,* et de NDM chez une souche de *Klebsiella pneumoniae.* Le délai de rendu de résultat était de 1 h 55 minutes en moyenne.

Notre étude a objectivé que les virus étaient incriminés dans la moitié des infections respiratoires aigües. La détection des virus par PCR multiplex contribue en cas de positivité à réduire l’usage inapproprié des antibiotiques. Par ailleurs, la PCR multiplex permet de détecter sept gènes de résistance bactérienne limitant ainsi l’utilisation d’antibiothérapie à large spectre en milieu de réanimation.

# Research article: Apport de l’équipe mobile de maladies infectieuses dans l’évaluation et l’adaptation de la prescription des anti-infectieux

**Published:** 2025-11-09

**Authors:** R. Jouirou, R. Bougossa, A. Ben Mabrouk, J. Chelli, F. Larbi

**Affiliations:** Service d’endocrinologie et de médecine interne, CHU Tahar Sfar, Mahdia, Tunisie

L’utilisation abusive et inappropriée d’antibiotiques a conduit à l’émergence de bactéries multirésistantes (BMR), d’où l’intérêt d’une équipe mobile de maladies infectieuses dans l’évaluation et l’adaptation de prescription des anti-infectieux, afin de rationaliser leur usage et préserver leur efficacité.

L’objectif de ce travail est de décrire les activités de l’équipe mobile de maladies infectieuses, et son implication dans l’évaluation et l’adaptation de prescription des anti-infectieux.

Il s’agit d’une étude rétrospective descriptive, incluant les avis de maladies infectieuses sollicités au CHU Tahar Sfar Mahdia entre août 2024 et février 2025. Une fiche de renseignements était remplie à chaque avis sollicité au cours de l’activité journalière, en dehors de l’activité des gardes et du service des urgences, et saisie en rétrospectif sur une base de données informatisée anonyme.

Durant la période d’étude, 206 avis étaient sollicités. Les services chirurgicaux étaient les plus demandeurs d’avis (n=167; 81,6%), particulièrement les services d’orthopédie et de chirurgie générale (38,8% et 25,2% respectivement). Deux-cent-un avis étaient sollicités pour aide à la prescription d’antibiothérapie, trois pour aide au diagnostic et deux pour les mesures préventives. La majorité des appels à propos d’anti-infectieux concernait des patients ayant un traitement antimicrobien en cours (n=160; 78,7%), dont la moitié étaient sous association d’antibiotique (n=105; 52%). Les anti-infectieux en cours appartiennent principalement aux familles des céphalosporines (n=54; 40,9%), pénicillines-inhibiteurs de bêta-lactamases (n=51; 38,6%), fluoroquinolones (n=37; 28%) et glycopeptides (n=22; 19,7%). Les infections communautaires représentaient le motif d’appel le plus fréquent (n=115; 56,4%). Parmi les infections associées aux soins, les infections du site opératoire étaient dominantes (n=73/89; 83%) en particulier les infections ostéo-articulaires sur matériel (n=33/89; 73,1%). Les infections étaient documentées dans 52% des cas (n=105), polymicrobiennes dans 25,9% (n=28). Les BMR représentaient% des bactéries isolées (n=30), dominées par les entérobactéries (n=14; 46,7%) et les bacilles à Gram négatif non fermentant (n=12; 40%). La décision thérapeutique était le changement d’antibiothérapie dans 139 cas (69,2%). Les motifs de changement étaient une adaptation aux données microbiologiques (n=43; 30,9%), un échec clinique (n=27; 19,4%) et un passage à la voie orale (n=21; 15,1%). La prescription s’est basée sur une l’administration d’antibiotique dans 118 cas (58,7%), et associait des céphalosporines (n=70; 34,7%), des fluoroquinolones (n=58; 28,7%) et les carbapénèmes (n=50; 24,8%).

L’aide au traitement ainsi que la prescription d’antibiotiques étaient les demandes les plus sollicitées. Les infections associées aux soins étaient fréquentes et le taux des BMR était alarmant, d’où l’intérêt d’un usage rationnel des antibiotiques.

# Research article: Antimicrobial Stewardship Interventions In Haematology Patients

**Published:** 2025-11-09

**Authors:** R. Rezgui, I. Chalbaoui, H. Ghedira, Z. Denden, M. Riden, R. Abid, F. Msaddak, S. Hannachi, R. Battikh

**Affiliations:** 1 Infectious disease department, Military Hospital of Tunis; 2 Clinical Haematology department, military hospital of Tunis

Hematology patients are at high risk of infection. Close coordination between infectious disease specialists and hematologists can optimize treatment outcomes for infectious complications in a hematology setting.

This study aimed to describe various antimicrobial stewardship (AMS) interventions within a hematology ward of a teaching hospital.

This prospective descriptive study examined all AMS interventions in the hematology ward of the main military teaching hospital in Tunis from January 2023 to February 2025.

A total of 325 AMS interventions were recorded. The median age of the included patients was 45.5 years (interquartile range [IQR] = 57-22). A gender ratio of 1.28 was observed. Patients were admitted for hematological malignancies in 87.4% of cases (n = 284), hemoglobinopathies in 10.5% of cases (n = 34), and primary immunodeficiencies in 2.2% of cases (n = 7). Of the interventions, 11.7% (n = 38) involved stem cell graft recipients. The corresponding physicians were trainees in 97.23% of cases (n = 316). Febrile neutropenia accounted for 177 (54.46%) of the AMS interventions. Antimicrobial treatment was prescribed in all cases, after receiving prior approval from an infectious disease specialist. The AMS team initiated antimicrobial therapy in collaboration with the relevant physicians in 24% of cases (n = 78). The chosen therapy, dosage, and treatment duration were adapted in 46.76% of cases (n = 152). Treatment escalation was withheld in 32.3% of cases (n = 105). Anti-infective therapy remained empirical following 68.9% of AMS and diagnostic stewardship procedures (n = 224).

Effective communication between infectious disease physicians and treating doctors, coupled with antimicrobial stewardship interventions, is pivotal in ensuring appropriate antimicrobial therapy for hematology patients.

# Research article: Conformité de l’antibiothérapie probabiliste: quel impact sur la mortalité et la durée d’hospitalisation?

**Published:** 2025-11-09

**Authors:** K. Cherif, M. Ben Selma, L. Tilouche, N. Ben Lasfar, H. Knanil, S. Boughattas, S. Ketata, A. Trabelsi, W. Hachfi

**Affiliations:** 1 Service de maladies infectieuses, CHU Farhat Hached, Sousse, Tunisie; 2 Laboratoire de microbiologie, CHU Sahloul, Sousse, Tunisie; 3 Université du Centre, Faculté de médecine de Sousse, Tunisie

Le bon usage des antibiotiques s’inscrit dans le cadre du programme *d’antimicrobial stewardship* afin d’améliorer la prise en charge en charge des pathologies infectieuses et la lutte contre l’antibiorésistance.

L’objectif de notre étude était d’évaluer la conformité de l’antibiothérapie probabiliste chez les patients adultes admis dans un CHU chirurgical et son impact sur la mortalité et la durée d’hospitalisation. Étude prospective, observationnelle et analytique, sur 3 mois du 1^er^ avril au 30 juin 2023, au laboratoire de microbiologie incluant tous les patients hospitalisés chez qui on avait prescrit une antibiothérapie. Le référentiel utilisé s’appuyait sur les recommandations de la Société de pathologie infectieuse de langue française. La conformité des prescriptions a été évaluée par un infectiologue aidé sur la base des critères suivants: l’indication, le choix et les modalités du traitement antibiotique (posologie et voie d’administration).

Au total, 378 patients ont été inclus. Il s’agissait d’un état de choc septique dans 17,5% des cas. L’infection était associée aux soins dans 52,4% des cas. L’antibiothérapie probabiliste consistait en une bithérapie dans 44,8% des cas et une trithérapie dans 9,9% des cas. Elle était conforme à l’antibioguide dans 51,5% des cas. La principale cause de non-conformité était une molécule inadéquate dans 178 des cas. Selon le site infectieux suspecté, le taux de conformité de l’antibiothérapie variait de 85,7% pour les infections de la sphère ORL à 25% pour les infections ostéo-articulaires. On a noté 52 cas de décès dont 65,4% avaient reçu une antibiothérapie probabiliste non conforme. L’étude analytique de l’impact de l’antibiothérapie probabiliste conforme sur la mortalité avait retrouvé qu’en l’absence de sepsis, tous les décès (4/4) sont survenus chez des patients ne recevant pas une antibiothérapie conforme avec un taux de survie à la sortie de 44% *versus* 56% en cas d’antibiothérapie conforme (p=0,025). En cas de sepsis, le taux de mortalité était plus élevé en cas d’antibio thérapie non conforme, 61,5% *versus* 38,5% en cas d’antibiothérapie conforme, de manière non significative (p=0,304). En cas de choc septique, le taux de mortalité était plus élevé avec une antibiothérapie probabiliste non conforme (62,9% *versus* 37,1%), sans différence significative (p=0,896).

Les recommandations n’ont été correctement appliquées que dans 51,5%. Une sensibilisation, formation et évaluation continue des pratiques des cliniciens est primordiale pour réduire l’usage inapproprié des antibiotiques.

# Research article: Caractérisation génique par puces ADN des bacilles à Gram négatif résistants isolés au centre de traumatologie et des grands brûlés

**Published:** 2025-11-09

**Authors:** S. Dhraief, R. Ammar, I. Chagra, L. Thabet

**Affiliations:** Laboratoire de biologie médicale, Centre de traumatologie et des grands brûlés, Université Tunis el Manar, Faculté de médecine de Tunis, UR22SP03, Tunisie

La détection des gènes de résistance est essentielle pour connaître l’épidémiologie locale, guider le traitement antimicrobien et limiter la propagation des bactéries multirésistantes. Objectif: Identifier les gènes de résistance aux antibiotiques chez les bacilles à Gram négatif (BGN) résistants aux carbapénèmes isolés au Centre de traumatologie et des grands brûlés.

Il s’agit d’une étude rétrospective sur 3 mois (avril-juin 2024) incluant tous les isolats cliniques de *Pseudomonas aeruginosa, d’Acinetobacter baumannii* et d’entérobactéries résistants aux carbapénèmes. La résistance aux carbapénèmes exclusivement due à une altération de la porine OprD a été exclue. L’identification bactérienne et l’antibiogramme ont été faits selon les méthodes conventionnelles. La détermination de la concentration minimale inhibitrice (CMI) de la colistine a été effectuée par microdilution en milieu liquide (UMIC^®^, Biocentric). La détection des gènes de résistance a été réalisée par hybridation inverse sur puces ADN à l’aide du kit MDR Direct Flow Chip^®^ sur des colonies bactériennes. Au total, 46 BGN résistants aux carbapénèmes ont été analysés chez 44 patients. Quarante souches (87%) étaient productrices d’une ou de plusieurs carbapénémases. Les métallo-β-lactamases bla NDM étaient les plus fréquentes (53,8%), suivies de bla OXA48-like (17,3%). Chez *P. aeruginosa*, les gènes de carbapénémases bla GES (66,7%) et bla VIM (16,7%) étaient dominants. Chez *A. baumannii* (Ab), les gènes bla OXA23-like (70%), bla NDM (40%) et bla OXA24-like (20%) étaient majoritaires. Le gène bla OXA51-like a été détecté chez toutes les souches d’Ab sauf une. Les entérobactéries portaient principalement bla NDM (82,8%) et bla OXA48-like (31%), ainsi que des BLSE bla SHV (58,6%), bla CTX-M (41,4%), bla DHA (27,6%) et bla CMY (13,8%). Un seul isolat de *Klebsiella pneumoniae* était résistant à la colistine (CMI=16mg/l), sans gène mcr détecté. Une souche d’A. *baumannii* portant le gène mcr-2 s’est révélée sensible à la colistine (CMI=0,5 mg/l). Notre étude a révélé une prévalence élevée des carbapénèmases, notamment bla NDM, chez les BGN résistants aux carbapénèmes. Ces résultats appellent à une vigilance accrue et à des mesures de prévention renforcées.

# Research article: Non-conformités analytiques en bactériologie: incidence et impacts

**Published:** 2025-11-09

**Authors:** S. Ben Jaafer, S. Frigui, Y. Chebbi, W. Achour

**Affiliations:** 1 Service des laboratoires, Centre national de greffe de moelle osseuse, Tunis, Tunisie; 2 Université Tunis El Manar, Faculté de médecine de Tunis, LR18ES39, Tunis, Tunisie

L’incidence des non-conformités (NC) analytiques représente un indicateur de qualité pour évaluer le processus analytique en bactériologie conventionnelle.

Nos objectifs étaient de déterminer l’incidence des NC analytiques en bactériologie et d’évaluer leur impact sur le délai de rendu des résultats et sur le coût des analyses.

Nous avons mené une étude descriptive, prospective et longitudinale incluant tous les prélèvements envoyés pour analyse bactériologique au Service des laboratoires du Centre national de greffe de moelle osseuse sur une période de 5 mois (juillet-novembre 2023). Une NC analytique a été définie par tout écart par rapport aux recommandations du référentiel en microbiologie médicale et du Comité de l’antibiogramme de la Société française de microbiologie.

Pour un total de 3 175 prélèvements inclus dans cette étude, ayant nécessité 5 917 opérations techniques, 180 NC analytiques ont été enregistrées. Ainsi, l’incidence des NC analytiques était de 56,7/1 000 prélèvements et de 30,4/1 000 opérations techniques. Le nombre de NC était corrélé aussi bien au nombre de prélèvements (R=0,564; p=0,006) qu’au nombre d’opérations techniques (R=0,471; p=0,027). Le responsable des NC analytiques était un médecin en formation dans 68% des cas. Les NC analytiques étaient dominées par « une mise en culture incorrecte » (28,9%), « une erreur dans l’étude de la sensibilité aux antibiotiques » (25%) et « la non-réalisation d’une opération technique » (20%). Les causes des NC étaient dominées par le manque de formation (28,1%), la distraction (22,3%), la pression temporelle (15,1%) et les préoccupations personnelles (13,7%). Les NC analytiques avaient un impact sur le délai de rendu des résultats dans 56,7% des cas (un jour de retard dans tous les cas) et sur le coût des analyses en termes de réactifs additionnels, dans 42,8% des cas, estimé à 1 056 dinars tunisiens de surcoût pendant la période d’étude. L’incidence élevée des NC analytiques en bactériologie est étroitement liée au nombre de prélèvements et d’opérations techniques, avec un impact sur le délai de rendu des résultats et le coût des analyses.

# Research article: Intérêt du panel BioFire^®^ FilmArray^®^ Blood Culture Identification (BCID2) dans le diagnostic des bactériémies et des méningites nosocomiales en milieu de réanimation

**Published:** 2025-11-09

**Authors:** O. Gargouri, S. Ben Brahim, K. Bouchaala, C. Ben Hamida, M. Bahloul, H. Karray

**Affiliations:** 1 Laboratoire de microbiologie CHU Habib Bourguiba de Sfax, Tunisie; 2 Service de réanimation CHU Habib Bourguiba de Sfax, Tunisie

L’utilisation des panels de diagnostic moléculaire offre une approche diagnostique syndromique et une adaptation précoce de l’antibiothérapie. Conçu pour détecter les bactériémies, le panel BioFire FilmArray Blood Culture Identification (P-BCID2) a également été employé par certaines équipes pour le diagnostic des méningites nosocomiales.

L’objectif du travail est d’évaluer l’apport du P-BCID2 (Biomérieux) dans la prise en charge des bactériémies et des méningites nosocomiales en le comparant à la culture conventionnelle.

Il s’agit d’une étude rétrospective incluant 33 patients hospitalisés au service de réanimation du CHU Habib Bourguiba de Sfax et ayant bénéficié du P-BCID2 conjointement à la culture conventionnelle. L’étude de la sensibilité aux antibiotiques a été réalisée selon les normes du Comité de l’antibiogramme de la Société française de microbiologie.

Trente-trois patients ont été inclus dans cette étude, avec un sex-ratio de 1,8. Les critères d’inclusion étaient une suspicion de méningite nosocomiale décapitée ou une instabilité hémodynamique au moment de la positivité des flacons d’hémocultures. Le P-BCID2 était positif pour 9/10 (89%) des cas de bactériémies étudiées. Une hémoculture était positive à *Corynebacterium* sp., bactérie non incluse dans P-BCID2. Pour les 8 autres cas, P-BCID2 a permis de détecter 11 bactéries dont 8 correspondant à de vraies bactériémies, et 3 bactéries provenant de flacons d’hémocultures contaminés. Le résultat fourni par P-BICD2 a permis d’adapter précocement l’antibiothérapie dans 57% des cas. Les gènes de résistance et la résistance phénotypique étaient concordants pour 7 parmi les 8 bactéries étudiées (87%). Parmi les 22 liquides cérébrospinaux (LCS), le P-BICD2 a permis de détecter 7 bactéries (32%): *Pseudomonas aeruginosa* (n=2), *Acinetobacter baumannii* (n=1), *Klebsiella pneumoniae* (n=1), *Streptococcus pneumoniae* (n=1), *Enterococcus faecium* (n=1) et *Streptococcus* sp. (n=1) (isolé d’empyème cérébral). Chez 5 patients, la bactérie n’a pas été détectée par culture. Le P-BCID2 était négatif pour 15 LCS pour lesquels la culture a été aussi négative pour 14 cas et positive à *Corynebacterium* sp. pour le dernier isolé d’une dérivation ventriculaire (soit une sensibilité de 93%). Le résultat de P-BCID2 a permis une adaptation de l’antibiothérapie à type d’escalade thérapeutique pour 2 des 7 LCS. Malgré son coût élevé, nos résultats montrent que le P-BCID2 semble être un outil utile pour le diagnostic rapide des bactériémies et des méningites nosocomiales en réanimation permettant une prise en charge précoce et adéquate.

# Research article: Purpura fulminans chez l’enfant tunisien: profil bactériologique et épidémiologique

**Published:** 2025-11-09

**Authors:** S. Boutabba, K. Meftah, S. Mhimdi, A. Miraoui, F. Aloui, Y. Chelbi, A. Bouafsoun, K. Menif, H. Smaoui

**Affiliations:** 1 Laboratoire de microbiologie, Hôpital d’enfants Béchir Hamza de Tunis, Université Tunis El Manar, Faculté de médecine de Tunis, LR18ES39, Tunis, Tunisie; 2 Unité de soins intensifs pédiatriques, Hôpital d’enfants Béchir Hamza de Tunis, Tunisie

Le purpura fulminans septique (PF) est une urgence grave, souvent causée par *Neisseria meningitidis* (*Nm*) ou d’autres bactéries. Il se caractérise par une septicémie sévère, qui peut évoluer en choc septique, défaillance multiviscérale et purpura nécrotique lié à la coagulation intravasculaire disséminée (CIVD). Cette étude vise à analyser les caractéristiques épidémiologiques, microbiologiques et pronostiques des cas de PF chez les enfants.

Une étude rétrospective descriptive a été menée de janvier 2017 à décembre 2024 au laboratoire de microbiologie du Centre hospitalier Béchir Hamza de Tunis. Tous les cas de purpura fulminans confirmés bactériologiquement chez des enfants âgés de 1 jour à 15 ans ont été inclus. Les hémocultures et les cultures de liquide cérébro-

spinal (LCS) ont été analysées selon le référentiel de microbiologie médicale. L’identification bactérienne a été réalisée à l’aide de méthodes classiques. La PCR en temps réel pour la détection de Nm, *Streptococcus pneumoniae (Sp)* et *Haemophilus influenzae* a été réalisée sur le LCS et le sang, en cas de culture négative. Les données cliniques ont été extraites des dossiers médicaux. L’étude a inclus 22 enfants hospitalisés pour un purpura fulminans septique, représentant une incidence moyenne de 2,75 cas par an. L’âge médian des patients était de 1 an et 8 mois (allant de 42 jours à 7 ans), avec 12 garçons et 10 filles. L’analyse de la répartition saisonnière a révélé une distribution inégale des cas au cours de l’année, avec le moins de cas enregistrés durant l’été (3 cas; 14%). Un syndrome méningé a été rapporté dans 45% des cas. Un choc a été observé chez 31% des patients. Aucune déficience immunologique connue n’a été identifiée. Parmi les 22 patients inclus, la culture (sang ou LCS) a été positive dans un seul cas. Le diagnostic reposait principalement sur la PCR, qui a permis d’identifier l’agent pathogène dans tous les autres cas. *Nm* a été identifié dans 95% des cas, avec une prédominance du sérogroupe B (59% des cas), suivi par le sérogroupe Y (18%). *Sp* a été identifié chez un patient. Aucun des patients n>était vacciné contre le méningocoque. En ce qui concerne les résultats, 68% des patients ont été hospitalisés en unité de soins intensifs pédiatriques, et le taux de mortalité observé était de 41% (le pronostic était inconnu pour quatre patients).

Le purpura fulminans est une urgence pédiatrique grave avec un taux de mortalité élevé. Dans cette étude, *Nm,* principalement le sérogroupe B, est l’agent causal. La PCR est supérieure à la culture, notamment après traitement antibiotique.

# Research article: Profil épidémiologique et bactériologique des méningites bactériennes communautaires chez l’enfant: avant et après vaccination antipneumococcique

**Published:** 2025-11-09

**Authors:** S. Lachiheb, N. Mseddi, K. Meftah, S. Mhimdi, M. Gantassi, A. Bouafsoun, H. Smaoui

**Affiliations:** Laboratoire de microbiologie, Hôpital d’enfants Béchir Hamza de Tunis, Tunisie

Les méningites bactériennes (MB) représentent une préoccupation majeure de santé publique, qui engage le pronostic vital avec un risque de séquelles graves. L’épidémiologie des MB est en évolution continue, suite au programme de vaccination.

Le but de cette étude est de décrire l’évolution du profil épidémiologique et bactériologique des MB communautaires chez l’enfant avant et après l’introduction du PCV10 dans le calendrier vaccinal national.

Il s’agit d’une étude rétrospective descriptive menée sur 3 périodes: P1 (pré vaccinale: janvier 2014-avril 2019, P2 (post vaccinale: janvier 2020-décembre 2024) et P3 (mise en place: mai-décembre 2019) au laboratoire de microbiologie de l’Hôpital d’enfants Béchir Hamza de Tunis. Ont été inclus tous les cas de MB communautaires, confirmées bactériologiquement (par culture ou PCR du LCS). L’identification bactérienne a été effectuée selon les méthodes conventionnelles. Une PCR sur liquide cérébrospinal a été réalisée en cas de culture négative. Le sérotypage a été réalisé par des techniques de PCR. L’étude de la sensibilité aux antibiotiques a été réalisée selon les recommandations du Comité de l’antibiogramme de la Société française de microbiologie.

Durant la période d’étude, 218 cas de MB ont été identifiés. L’âge médian était de 12 mois [1 mois à 15 ans]. Le sex-ratio (H/F) était de 1,27. Le diagnostic était confirmé par culture dans 173 cas. *Streptococcus pneumoniae (Sp)* était le germe le plus fréquemment identifié (56%) suivi de *Neisseria meningitidis* (26%) et *Haemophilus influenzae* (9%). La diminution des cas de MB à *Sp* était non significative entre P1 et P2 (p=0,7). En P1,88% des enfants atteints de MB à *Sp* étaient âgés de moins de 5 ans tandis qu’en P2,57% étaient âgés de plus de 5 ans (p<0,00001). Une augmentation des sérotypes non-vaccinaux (SNV PCV10) a été notée en P2 (15/23) (p=0,003). Le taux de *Sp* de sensibilité diminuée aux pénicillines est passé de 66% en P1 à 87% en P2 (p=0,03).

*Sp* demeure l’agent majeur des MB chez l’enfant de plus de 5 ans avec une expansion des souches non vaccinales et une augmentation des taux de résistances aux antibiotiques après l’introduction du PCV10 en Tunisie.

# Research article: Extended Spectrum Beta-lactamase producing *Shigella sonnei* nationwide outbreak likely linked to unsafe water sanitation practices, Tunisia, 2022/2023

**Published:** 2025-11-09

**Authors:** S. Dhaouadi, F. Ben Youssef, E. Mziou, A. Hechaichi, H. Letaief, M. Safer, R. Mhadhbi, F. Larouchi, S. Jelassi, S. El Mili, H. Smaoui, A. Hammami, S. Guermazi, M. Rebhi, R. Ben Aissa, H. Bouguerra, N. Bouafif Ep Ben Alaya

**Affiliations:** 1 National Observatory of New and Emerging Diseases, Tunis, Tunisia; 2 Mediterranean and Black Sea Programme in Intervention Epidemiology Training (MediPIET), European Centre for Disease Prevention and Control (ECDC), Solna, Sweden; 3 Faculty of Medicine Tunis, University Tunis El Manar, Tunisia; 4 Laboratory of Microbiology, Children’s Hospital Béchir Hamza of Tunis, Tunis, Tunisia; 5 Laboratory of Microbiology, Habib Bourguiba University Hospital, Tunisia; 6 National Authority for the Sanitary Safety of Food Products, Tunis, Tunisia; 7 Reference laboratory of Shigellosis, Water and food control laboratory Pasteur Institute of Tu-nis, Tunisia; 8 Tunisian Field Epidemiology Training Programme (T-FETP), Tunis, Tunisia; 9 Faculty of Medicine of Sfax, Tunisia; 10 Tunisian FETP, Eastern Mediterranean Public Health Network (EMPHNET), Amman, Jordan

On November 3, 2022, the reference laboratory confirmed 60 cases of *Shigella sonnei* producing extended-spectrum beta-lactamases among hospitalized children.

National and regional multisectoral outbreak response teams began investigating to identify the source of the outbreak and implement appropriate measures.

A suspected case was defined as a Tunisian resident exhibiting gastrointestinal symptoms for which no other cause was identified. A confirmed case was defined as a suspected case in which S. *sonnei* was isolated on or after June 1, 2022. We actively searched for cases by interviewing patients and reviewing laboratory and medical records. We inspected the sanitary conditions of the households of the index cases and collected environmental samples. We calculated the attack rate (AR) of suspected cases per 100,000 inhabitants, as well as the case fatality ratio (CFR), which is defined as the number of deaths among confirmed cases.

As of June 30, 2023, 653 suspected cases (AR = 5.5) had been reported. Of these, 378 (58%) were confirmed, and one death occurred (CFR = 0.2%). The epi-curve revealed an initial common source, followed by human-to-human transmission. A peak occurred in week 48 of 2022 with 74 cases, after which the number of cases decreased. The AR was highest among children.

This outbreak was likely caused by drinking contaminated water and failing to comply with basic hygiene practices. Due to the emergence of ESBL-producing S. *sonnei*, we recommend strengthening microbiological surveillance and maintaining multisectoral collaboration in public health.

# Research article: Profil bactériologique des bactériémies au service de réanimation des brûlés au Centre de traumatologie et des grands brûlés (2020-2024)

**Published:** 2025-11-09

**Authors:** Y. Ben Chehida, S. Dhraief, E. Thabet, H. Fredj, A. Mokline, L. Thabet

**Affiliations:** 1 Laboratoire de biologie médicale, Centre de traumatologie et des grands brûlés, Université Tunis el Manar, Faculté de médecine de Tunis, UR22SP03, Tunisie; 2 Service de réanimation des brûlés, Centre de traumatologie et des grands brûlés, Tunis, Tunisie

La prise en charge des bactériémies pose un problème majeur, surtout chez les patients immunodéprimés, notamment les grands brûlés.

Le but de cette étude est de décrire le profil bactériologique et la sensibilité aux antibiotiques des bactéries isolées dans les hémocultures réalisées au service de réanimation des brûlés.

Il s’agit d’une étude descriptive rétrospective réalisée au laboratoire de biologie du Centre de traumatologie et des grands brûlés, portant sur toutes les souches isolées dans les hémocultures positives provenant du service de réanimation des brûlés sur une période de cinq ans (2020-2024). L’automate BD Bactec™ FX a été utilisé pour la mise en culture des hémocultures. L’identification bactérienne a été faite selon les méthodes conventionnelles. L’étude de la sensibilité aux antibiotiques a été réalisée et interprétée selon les normes du Comité de l’antibiogramme de la Société française de microbiologie, annuellement révisées.

Les bactériémies représentaient 29,5% de l’ensemble des infections observées en réanimation des brûlés. Sur un total de 1 800 souches bactériennes isolées, les principales espèces étaient *Klebsiella pneumoniae* (9,7%), *Staphylococcus epidermidis* (8,9%), *Acinetobacter baumannii* (8,9%), *Pseudomonas aeruginosa* (6,8%), *Providencia stuartii* (6,5%) et *Staphylococcus aureus* (6,1%). Concernant *K. pneumoniae*, les taux de résistance étaient de 80,1% au céfotaxime, 53% à l’ertapénème, 81,7% à la ciprofloxacine et 76,6% à la gentamicine. Le taux de *K. pneumoniae* productrices de bêta-lactamases à spectre étendu a diminué de 29,3% en 2020 à 20,7% en 2024. Vingt-six souches étaient résistantes à la tigécycline et cinq à la colistine. La méticillinorésistance était plus fréquente chez S. *epidermidis* (54,8%) que chez S. *aureus* (27,9%) chez qui elle était fluctuante durant les cinq années de la période d’étude, oscillant entre 16,7% et 41,2%. Aucune résistance aux glycopeptides ni à la tigécycline n’a été détectée chez *Staphylococcus spp. A. baumannii* et *P. aeruginosa* présentaient des taux de résistance de 92,7% et 59,7% à la ceftazidime, 89,5% et 69,9% à l’imipénème, 82,8% et 70,4% à l’amikacine, 91% et 56,2% à la ciprofloxacine, respectivement. Une seule souche d’A. *baumannii* était résistante à la colistine. Toutes les souches de *P. aeruginosa* étaient sensibles à cet antibiotique. Concernant *Providencia stuartii,* les taux de résistance étaient de 55,5% à la ciprofloxacine, 89,9% à la céfotaxime, 45,9% à l’amikacine, 28,6% à l’ertapénème et 43,1% à la fosfomycine.

En conclusion, notre étude a montré une multirésistance aux antibiotiques chez les patients admis en réanimation des brûlés imposant une surveillance épidémiologique régulière de l’écologie bactérienne et l’application stricte des mesures d’hygiène.

# Research article: Apport de la génomique, la bioinformatique et l’intelligence artificielle dans la prédiction de la résistance aux antibiotiques chez *Streptococcus pneumoniae*

**Published:** 2025-11-09

**Authors:** F. Smaoui, B. Ksibi, N. Ben Ayed, S. Ktari, O. Gargouri, O. Gargouri, S. Bougharriou, S. Mezghani, B. Mnif, F. Mahjoubi, H. Karray, A. Hammami

**Affiliations:** Laboratoire de recherche MPH (LR03SP03), Laboratoire de microbiologie, CHU Habib Bourguiba, Sfax, Tunisie

*Streptococcus pneumoniae* est une cause majeure d’infections invasives graves qui constituent un enjeu de santé publique. Le séquençage génomique offre une nouvelle perspective pour surveiller la résistance aux antibiotiques chez *S. pneumoniae*, mais son interprétation reste un défi.

Notre objectif était d’évaluer la performance d’algorithmes bio-informatiques et d’intelligence artificielle pour prédire la résistance aux principaux antibiotiques à partir des données génomiques chez *S. pneumoniae.*

L’étude a porté sur 148 souches invasives de S. *pneumoniae* isolées durant la période 2012-2022 au laboratoire de microbiologie du CHU Habib Bourguiba de Sfax. La résistance phénotypique a été évaluée selon les recommandations du Comité de l’antibiogramme de la Société française de microbiologie. Le séquençage des génomes bactériens a été réalisé par technologie Illumina. Des modèles d’apprentissage automatique ont été appliqués pour prédire la résistance aux bêtalactamines à partir des séquences des gènes pbp2A-pbp2B-pbp2X. Pour les autres antibiotiques, des algorithmes bio-informatiques ont évalué la résistance en fonction de gènes et mutations spécifiques. La concordance avec les résultats phénotypiques a été évaluée par le taux d’erreurs très majeures, majeures et mineures.

Les modèles d’apprentissage automatique ont montré une concordance de 76% pour la pénicilline G, 80% pour l’amoxicilline et 61% pour le céfotaxime. La plupart des discordances était mineures, tandis que le taux combiné d’erreurs majeures et très majeures était inférieur à 1%. Concernant les autres antibiotiques, l’analyse bio-informatique des déterminants de résistance a montré une concordance parfaite pour l’érythromycine, le chloramphénicol et la lévofloxacine. Toutefois, des erreurs majeures et très majeures ont été observées pour la tétracycline (tetM détectée chez 24% des souches sensibles) et le triméthoprime-sulfaméthoxazole (mutations en folA/folP présentes chez 8% des souches sensibles), respectivement.

L’analyse bio-informatique des données génomiques, combinée à l’intelligence artificielle, offre une approche prometteuse pour la prédiction de la résistance aux antimicrobiens. Toutefois, des améliorations restent nécessaires pour limiter les discordances, notamment en intégrant des approches transcriptomiques et protéomiques.

# Research article: Emergence of Colistin-Resistant Enterobacterales in a Tunisian Hospital: Molecular Characterization and Resistance Mechanisms

**Published:** 2025-11-09

**Authors:** M. Ennaceur, S. Marzouki, M. Kaidi, M. Houari, M.A. Najeh, S. Chouaieb

**Affiliations:** 1 Bacteriology Unit, Laboratory Department, Habib Thameur Hospital, Tunis, Tunisia; 2 Laboratory Department, Habib Thameur Hospital, Tunis, Tunisia

Data on plasmid-mediated colistin resistance in clinical bacteria remain limited in Tunisia. This study aimed to assess the epidemiology of colistin resistance and the dissemination of the mcr gene in clinical Enterobacterales collected from our hospital.

A cross-sectional study was conducted in 2024 on colistin-resistant Enterobacterales isolates. Molecular characterization, including the detection of the mcr genes and β-lactamase resistance genes, was performed using PCR (DNA flow technique).

Fourteen colistin-resistant *Klebsiella pneumoniae (KP)* strains were identified, with minimum inhibitory concentration values ranging from 4 to 64 mg/L. Most were isolated from the emergency and intensive care unit (9/14, 64%), followed by the anesthesia and intensive care (3/14, 21%), urology (1/14, 7%), and internal medicine (1/14, 7%). The most frequent sample types were respiratory specimens (9/14, 64%), urine (3/14, 21%), and blood cultures (2/14, 14%). Regarding resistance genes, 10/14 isolates (71%) harbored the extended-spectrum beta-lactamase gene blaCTX-M, and 6/14 (43%) carried blaSHV. Four isolates (28%) carried blaOXA-48, two isolates (14%) carried blaNDM, and eight isolates (57%) co-harbored both and exhibited carbapenem resistance. The mcr-1 gene was detected in a single *KP* isolate that carried both blaSHV and blaOXA-48.

This study underscores the importance of routine colistin susceptibility testing in Enterobacterales isolates and molecular screening for resistance mechanisms to guide appropriate antimicrobial stewardship.

# Research article: Profil bactériologique et sensibilité aux antibiotiques des bactéries responsables des infections liées aux cathéters

**Published:** 2025-11-09

**Authors:** W. Lazreg, Y. Maatouk, M. Farhat, A. Kahloul, S. Miri, A. Belghouthi, R. Bziouech, J. Boukadida, M. Marzouk, N. Hannachi

**Affiliations:** Laboratoire de microbiologie, CHU Farhat Hached Sousse, Tunisie

Les infections liées aux cathéters demeurent une cause importante d’infections nosocomiales. Elles peuvent entraîner des conséquences cliniques sévères, allant de l’infection locale à la septicémie. Elles sont souvent causées par des agents pathogènes résistants aux antibiotiques, ce qui rend leur prise en charge complexe. L’objectif de ce travail est d’évaluer le profil bactériologique et d’étudier la sensibilité aux antibiotiques des bactéries responsables d’infections liées au cathéter (ILC).

Nous avons réalisé une étude rétrospective descriptive sur une période de 4 ans (20212024) ayant inclus tous les cas d’ILC chez les patients hospitalisés dans notre établissement. La méthode de Brun-Buisson a été appliquée pour identifier les infections, et l’évaluation de la sensibilité aux antibiotiques a été effectuée conformément aux recommandations du Comité de l’antibiogramme de la Société française de microbiologie.

Durant l’étude, 437 souches bactériennes ont été isolées, la majorité étant des bacilles à Gram négatif (BGN) (56%). Parmi celles-ci, les entérobactéries prédominaient (163; 66%), avec en tête de liste *Klebsiella pneumoniae* (59,5% des entérobactéries). *Acinetobacter baumannii* était le BGN non fermentaire le plus commun, représentant 20,6% des BGN. Concernant les cocci à Gram positif, les staphylocoques à coagulase négative (SCN) étaient prépondérants (75,6%). Le service de néonatologie était le plus pourvoyeur d’ILC (33,2%), suivi des services de réanimation médicale (25,6%) et chirurgicale (8,2%). Les entérobactéries présentaient un taux de production de bêta-lactamases à spectre élargi de 18,4% et une résistance de 27% à l’ertapénème. Elles étaient résistantes dans 45,3% des cas aux fluoroquinolones et dans 39,2% à la gentamicine. Concernant *A. baumannii*, toutes les souches étaient hautement résistantes, sensibles uniquement à la colistine. Quant aux SCN, 59,6% étaient résistants à la méticilline et 15,3% aux glycopeptides. L’émergence des résistances bactériennes dans les ILC souligne l’urgence d’adapter les protocoles thérapeutiques et de promouvoir des alternatives innovantes. Une surveillance continue des agents pathogènes en cause et de leur profil de résistance semble nécessaire pour une prise en charge appropriée.

# Research article: Profils épidémiologiques et bactériologiques des souches *d’Acinetobacter baumannii* isolées au CHU Fattouma Bourguiba de Monastir

**Published:** 2025-11-09

**Authors:** G. Guedri, O. Gargouri, Y. Kadri, M. Mastouri

**Affiliations:** 1 Laboratoire de microbiologie de CHU Habib Bourguiba, Sfax, Tunise; 2 Laboratoire de microbiologie de CHU Fattouma Bourguiba, Monastir, Tunisie

*Acinetobacter baumannii* est un pathogène nosocomial multirésistant qui provoque des infections sévères. Cette étude a été menée au CHU Fattouma Bourguiba de Monastir (CHU FBM). L’objectif était de déterminer le profil épidémiologique et l’antibiorésistance des souches d’A. *baumannii* isolées au laboratoire de microbiologie du CHU FBM sur 10 ans.

Il s’agit d’une étude descriptive rétrospective, portant sur toutes les souches non redondantes dA. *baumannii* isolées à partir de prélèvements cliniques chez les malades hospitalisés au CHU FBM, de janvier 2014 à décembre 2023. L’identification bactérienne a été effectuée par les techniques conventionnelles et l’étude de la sensibilité aux antibiotiques selon les recommandations du Comité de l’antibiogramme de la Société française de microbiologie.

Durant la période d’étude, un total de 1 268 souches d’A. *baumannii* a été inclus. La quasi-totalité des souches provenaient du milieu hospitalier. Les services les plus pourvoyeurs étaient la réanimation chirurgicale (46,2%), la réanimation médicale (23,5%) et la chirurgie générale (5,9%). Les principaux sites d’isolement étaient les prélèvements respiratoires (54,5%), les prélèvements de pus (15,6%) et les hémocultures (9,8%). La résistance aux antibiotiques a été très élevée: ticarcilline (94,5%), piperacilline (94,9%), piperacilline-tazobactam (92,9%), ceftazidime (92,1%), imipenème (88,1%), gentamicine (86,4%) et rifampicine (55,2%). Toutefois, les taux de résistance à la colistine étaient très faibles (1,5%). La résistance d’A. *baumannii* aux antibiotiques évolue de manière continue. Les taux de résistance observés au cours des dix années de l’étude montrent une augmentation constante. La résistance à l’imipénème est passée de 79,4% en 2014 à 92,2% en 2023. Les taux de résistance aux antibiotiques étaient particulièrement élevés dans les unités de soins intensifs (USI) pour presque tous les antibiotiques. Ainsi, le taux de résistance à l’imipénème en USI atteignait environ 90,5%.

Le taux élevé d’A. *baumannii* multirésistant, en particulier en USI, nécessite une surveillance rigoureuse, des mesures strictes d>hygiène et d>asepsie, ainsi qu>une utilisation rationnelle des antibiotiques pour limiter sa propagation.

# Research article: Third-generation cephalosporin-resistant Enterobacteria: prevalence and resistance profile

**Published:** 2025-11-09

**Authors:** I. Ghoufa, Y. Maatouk, M. Farhat, H. Miladi, A. Kahloul, S. Miri, D. Rouis, J. Fathallah, J. Boukadida, N. Hannachi, M. Marzouk

**Affiliations:** Service de microbiologie, CHU Farhat Hached de Sousse, Tunisie

The increasing prevalence of Enterobacteriaceae resistant to third-generation cephalosporins (3GC-RE) poses a growing threat to public health, particularly in healthcare settings. This resistance complicates infection management, increases mortality, and raises healthcare costs. This study aimed to describe the epidemiology of 3GC-RE and assess their resistance profile. This retrospective descriptive study was conducted over four years (2021-2024) and included all 3GC-RE isolates collected in the microbiology department. Bacterial identification was performed using conventional methods. Antibiotic susceptibility was determined in accordance with CA-SFM/EUCAST recommendations.

A total of 1,723 3GC-RE isolates were collected. The study population was primarily adults (67%), with a slight female predominance (sex-ratio M/F = 0.77). The departments with the most isolates were the neonatology (15.8%), the emergency department (13.5%), pediatrics (11.1%), and the intensive care unit (8.7%). The 3GC-RE isolates were primarily from urine (64.5%) and blood cultures (11.4%). The predominant species were *Klebsiella pneumoniae* (57.7%) and *Escherichia coli* (39.3%). The following resistance rates were observed: amoxicillin-clavulanic acid (88%), piperacillin-tazobactam (52%), imipenem (12.8%), ciprofloxacin (46.8%), gentamicin (47.5%), and trimethoprim-sulfamethoxazole (36.8%). Colistin was the most effective molecule, with a resistance rate of 5%.

This study reveals a high prevalence of 3GC-RE. Their association with other resistances complicates therapeutic choices and underscores the need to strengthen microbiological surveillance and implement strict strategies to limit the spread of resistance.

# Research article: Microbial profiles and antibiotic resistance trends in cerebrospinal fluid isolates from meningitis cases

**Published:** 2025-11-09

**Authors:** J. Mosbah, Y. Maatouk, A. Kahloul, S. Miri, F. Dallel, W. Ben Abid, H. Miladi, J. Boukadida, N. Hannachi, M. Marzouk

**Affiliations:** Service de microbiologie, CHU Farhat Hached de Sousse, Tunisie

Meningitis is a serious condition with a high mortality rate and the potential for severe, long-term complications. Microbiological analysis of meningitis and antibiotic resistance is critical for ensuring proper management and improving patient outcomes.

This study aimed to analyze bacterial isolates from cerebrospinal fluid (CSF) and evaluate their antibiotic susceptibility.

A retrospective study was conducted on nonredundant strains isolated from CSF over four

years (2021-2024). Data were collected using requisition forms and the laboratory register. Bacterial identification was performed using conventional techniques. Antibiotic susceptibility testing was performed according to recommendations from the Comité de l’antibiogramme de la Société française de microbiologie.

During the study period, 96 bacterial strains were isolated from CSF. The sex-ratio was 1.13, with a predominance of pediatric cases (66.6%). Most cases originated from the neonatology department (38.5%), followed by the adult emergency department (17.7%) and the pediatrics department (12.5%). The most frequently isolated bacterium was *Streptococcus pneumoniae* (23%), followed by *Klebsiella pneumoniae* (21%) and *Staphylococcus aureus* (10%). Three cases of *Neisseria meningitidis* infection and one case of *Haemophilus influenzae* infection were reported. *K. pneumoniae* predominated in the neonatal population, while S. *pneumoniae* was more prevalent in adults (p < 0.05). Decreased susceptibility to penicillin accounted for 37.5% of S. *pneumoniae* cases. Resistance rates to penicillin G and cefotaxime were 31% and 14.2%, respectively. Resistance rates to other antibiotic families varied: 40.9% for erythromycin, 33% for lincomycin, and 26.3% for tetracycline. No resistance to glycopeptides was reported. Extended-spectrum beta-lactamase production was observed in 53% of *K. pneumoniae* strains, and 20% exhibited hyperproduced cephalosporinase. Ten percent of *K. pneumoniae* strains exhibited carbapenem resistance. Nearly one-quarter of the S. *aureus* strains were resistant to methicillin.

This study highlights the predominance of *S. pneumoniae* and *K. pneumoniae* in meningitis cases in our region. The alarming resistance rates call for enhanced antimicrobial stewardship and alternative treatments. To combat rising antibiotic resistance in meningitis management, continuous surveillance and updated guidelines are essential.

# Research article: Étude comparative de la résistance aux antibiotiques chez *Staphylococcus aureus* au CTGB durant deux périodes (2013-2018 et 2019-2024)

**Published:** 2025-11-09

**Authors:** Y. Haddad, S. Dhraief, M. Ben Amor, L. Thabet

**Affiliations:** Laboratoire de biologie médicale, Centre de traumatologie et des grands brûlés, Université Tunis el Manar, Faculté de médecine de Tunis, UR22SP03, Tunisie

La résistance aux antibiotiques des infections dues à *Staphylococcus aureus* constitue un enjeu majeur en milieu hospitalier. La surveillance des profils de résistance permet d’adapter les stratégies thérapeutiques, d’anticiper les évolutions des souches bactériennes et d’optimiser l’efficacité des traitements antibiotiques.

Notre objectif était de comparer le profil de résistances des souches S. *aureus* au centre de traumatologie et des grands brûlés (CTGB) entre deux périodes (2013-2018=P1 et 2019-2024=P2). Nous avons réalisé une étude rétrospective menée sur les souches de S. *aureus* isolées à partir des prélèvements bactériologiques des patients hospitalisés au CTGB entre 2013 et 2024. L’identification bactérienne a été réalisée par les méthodes conventionnelles. L’étude de l’antibiorésistance a été réalisée conformément aux recommandations du Comité de l’antibiogramme de la Société française de microbiologie, annuellement révisées.

Au total, 3 121 souches de S. *aureus* ont été isolées pendant 12 ans, représentant 11,8% de l’ensemble des bactéries isolées au CTGB. En P1, les souches provenaient surtout des hémocultures (31,2%) et de prélèvements cutanés (26,7%). En P2, elles étaient principalement isolées à partir des prélèvements cutanés (23,1%), de pus (17,1%) et d’hémocultures (13,5%). La majorité des souches provenait des services de réanimation des brûlés (53,7% vs 36%), de l’orthopédie (15,2% vs 23,3%) et de l’anesthésie-réanimation (12% vs 11,3%). Les résistances des souches de *S. aureus* à la ciprofloxacine, clindamycine, fosfomycine, rifampicine, gentamicine et au sulfaméthoxazole-triméthoprime ont significativement diminué entre les deux périodes, tandis que celle à l’acide fusidique a significativement augmenté. Aucune différence significative n’a été montrée pour la résistance à la pénicilline G et à l’érythromycine. Dans notre série, toutes les souches de S. *aureus* étaient sensibles à la quinupristinedalfopristine, aux glycopeptides et au linézolide. Durant toute la période, 1 094 souches de S. *aureus* résistant à la méticilline (SARM) ont été identifiées (35%). Leur taux a significativement chuté de 46,4% en P1 à 24,5% en P2. Les souches de SARM montraient une résistance nettement plus élevée que celles de S. *aureus* sensibles à la méticilline (SASM). Les résistances des SARM à la ciprofloxacine, fosfomycine, rifampicine, gentamicine et au sulfaméthoxazole-triméthoprime ont significativement diminué entre P1 et P2. En revanche, celle de l’acide fusidique a montré une augmentation significative. Aucune différence significative n’a été observée pour l’érythromycine, clindamycine et tigécycline.

Notre étude a révélé une réduction notable de certaines résistances de S. *aureus* entre P1 et P2, probablement liée à l’application stricte des mesures d’hygiène et à une gestion plus rationnelle des antibiotiques dans notre centre.

# Research article: Infections ostéo-articulaires sur matériel au Centre de traumatologie et des grands brûlés (CTGB): souches bactériennes et résistance aux antibiotiques

**Published:** 2025-11-09

**Authors:** E. Thabet, S. Dhraief, Y. Haddad, H. Annabi, L. Thabet

**Affiliations:** 1 Laboratoire de biologie médicale, Centre de traumatologie et des grands brûlés, Université Tunis el Manar, Faculté de médecine de Tunis, UR22SP03, Tunisie; 2 Service de chirurgie orthopédique, Centre de traumatologie et des grands brûlés de Ben Arous, Tunisie

Les infections ostéo-articulaires sur matériel (IOAM) présentent un risque élevé d’échec thérapeutique et de morbidité à long terme, avec une évolution fréquente vers la chronicité ou la récidive. L’antibiothérapie est un pilier fondamental de la prise en charge des infections ostéoarticulaires (IOA). Une antibiothérapie adaptée est cruciale pour favoriser la guérison, éviter les complications, et limiter les risques de rechute. L’objectif de ce travail est de décrire le profil épidémiologique et le profil de résistance aux antibiotiques des principales souches bactériennes responsables d’IOAM.

Il s’agit d’une étude rétrospective, descriptive sur une période de six ans (2019-2024), réalisée au Centre de traumatologie et des grands brûlés de Tunis. Tous les prélèvements ostéo-articulaires provenant de patients avec suspicion clinique d’IOAM ont été inclus. L’identification des bactéries a été réalisée selon les méthodes conventionnelles. Les antibiogrammes ont été réalisés par diffusion sur milieu gélosé et interprétés selon les recommandations du Comité de l’antibiogramme de la Société française de microbiologie, annuellement révisées.

Sur 547 prélèvements faits, 770 souches bactériennes ont été isolées avec un taux de positivité de 80,4%. L’infection était polymicrobienne dans 196 cas (35,8%). *Staphylococcus aureus* était la bactérie la plus fréquemment isolée (22,2%), suivie de *Klebsiella pneumoniae* (15,1%), des staphylocoques à coagulase négative (SCN) (10,6%) et de *Pseudomonas aeruginosa* (9,8%). *Escherichia coli* et *Acinetobacter baumannii* représentaient 7.1% et 5,3% des souches isolées respectivement. Le taux de résistance à la méticilline était de 20,4% pour S. *aureus*, contre 54,8% pour les SCN. Toutes les souches de *Staphylococcus spp.* étaient sensibles aux glycopeptides, au linézolide et à la tigécycline. Concernant *K. pneumoniae,* les taux de résistance au céfotaxime, à la pipéracilline-tazobactam et à l’imipénème étaient de 63,2%, 42,7% et 15,3%, respectivement. Les souches de *K. pneumoniae* étaient résistantes à la ciprofloxacine et à la gentamicine dans 59,8% et 42,7% des cas, respectivement. Une seule souche était résistante à la colistine. *P. aeruginosa* présentait des résistances à la ceftazidime et à l’imipénème de l’ordre de 17,1% pour les deux antibiotiques. Aucune souche n’était résistante à la colistine. On note que 90,2% des souches d’A. *baumannii* étaient résistantes au ceftazidime, 87,8% à l’imipénème, 78% à la ciprofloxacine et 73.1% à la gentamicine. Aucune souche n’était résistante à la colistine.

Une surveillance rigoureuse des IOAM est essentielle pour orienter l’antibiothérapie probabiliste et améliorer le pronostic des patients.

# Research article: *Acinetobacter baumannii*: épidémiologie et profil de résistance aux antibiotiques au CTGB (2019-2024)

**Published:** 2025-11-09

**Authors:** M. Ben Amor, S. Dhraief, R. Ammar, L. Thabet

**Affiliations:** Laboratoire de biologie clinique, centre de traumatologie et des grands brûlés, Université Tunis el Manar, Faculté de médecine de Tunis, UR22SP03, Tunisie

*Acinetobacter baumannii* est un BGN non fermentaire, principalement responsable d’infections nosocomiales.

L’objectif de notre étude était de décrire le profil épidémiologique et de déterminer le profil de résistance aux antibiotiques des souches d’A. *baumannii* isolées au Centre de traumatologie et des grands brûlés (CTGB).

Il s’agit d’une étude descriptive rétrospective menée sur une période de six ans (2019-2024) incluant toutes les souches d’A. *baumannii* isolées au laboratoire du CTGB. L’identification bactérienne a été effectuée par les méthodes conventionnelles. La sensibilité aux antibiotiques a été évaluée selon les recommandations du Comité de l’antibiogramme de la Société française de microbiologie, annuellement révisées. La concentration minimale inhibitrice de la colistine a été déterminée par micro-dilution en milieu liquide (UMIC^®^, Biocentric).

Nous avons colligé 1 362 souches d’A. *baumannii* représentant 8,15% des bactéries isolées, 15,2% des BGN et 35,8% des BGN non fermentaires. La majorité des souches provenaient des services de réanimation des brûlés (62,6%) et d’anesthésie réanimation (17,3%). *A. baumannii* a été isolé essentiellement dans les hémocultures (22,5%), les prélèvements respiratoires (18,6%) et les cultures de cathéters (15,7%). *A. baumannii* se manifestait au CTGB selon un état endémique avec des pics épidémiques. Les souches présentaient une fréquence élevée de résistance à la pipéracillinetazobactam (94,5%), au ceftazidime (93,7%), à l’imipenème (91,6%), à la ciprofloxacine (92,6%) et à l’amikacine (88,5%). Treize souches d’A. *baumannii* (1%) étaient résistantes à la colistine. La prévalence inquiétante des souches multirésistantes d’A. *baumannii,* surtout dans les services de réanimation de notre centre, souligne l’importance de renforcer les mesures d’hygiène et de mettre en place une surveillance continue pour limiter la propagation de cette bactérie.

# Research article: Évaluation de l’intelligence artificielle de type *chain of thought prompting* dans la détection de mécanismes de résistance des Enterobacterales aux bêtalactamines

**Published:** 2025-11-09

**Authors:** I. Chagra, S. Dhraief, L. Thabet

**Affiliations:** Laboratoire de biologie médicale, Centre de traumatologie et des grands brûlés, Université Tunis el Manar, Faculté de médecine de Tunis, UR22SP03, Tunisie

L’intelligence artificielle (IA) apparaît comme un outil prometteur dans l’arsenal du microbiologiste pour l’interprétation des antibiogrammes. *Chain of thought (COT) prompting* est une nouvelle technologie dans le domaine de l’IA visant à améliorer la logique décisionnelle des modèles d’IA.

Ce travail a comme objectif d’évaluer la performance du *COT prompting* d’IA dans la détection des mécanismes de résistance des Enterobacterales aux bêtalactamines selon les recommandations du Comité de l’antibiogramme de la Société française de microbiologie de 2024.

Cette étude rétrospective compare quatre configurations d’IA pour l’interprétation de 200 antibiogrammes d’Enterobacterales. Deux systèmes ont été évalués: l’un basé sur la technologie *COT* (DeepSeek-R1-70b) et l’autre sans *COT* (Llama-3,3-70b), chacun testé avec ou sans les recommandations du Comité de l’antibiogramme de la Société française de microbiologie 2024. Les configurations évaluées incluent: A *(COT* + Recommandations), B *(COT* sans Recommandations), C (Non *COT* + Recommandations) et D (Non *COT* sans Recommandations). Chaque IA a estimé la probabilité de trois mécanismes de résistance (ampC, BLSE, carbapénémase), et les résultats ont été comparés à ceux d’un expert selon la sensibilité, spécificité, VPP, VPN et durée de réponse.

La configuration A a montré une spécificité plus élevée que la configuration D pour la détection des BLSE (86,5%), des ampC (94%) et des carbapénémases (97,4%). Sa sensibilité a augmenté pour les carbapénémases (100%) et les ampC (70,1%), mais a diminué pour les BLSE (70,3%). La configuration B a également amélioré la spécificité et la sensibilité pour les carbapénémases (90,4% et 88,6%), mais a entraîné une baisse de la sensibilité pour les ampC (59,7%) et les BLSE (41,9%). La configuration D avait tendance à générer des faux positifs pour les BLSE, avec une spécificité de seulement 9,5% et une sensibilité de 97,3%. La configuration A a montré les meilleures valeurs de VPP et VPN pour les trois mécanismes étudiés. Lors de l’évaluation des BLSE par espèce, elle a présenté une sensibilité et une spécificité élevées pour *E. coli* (100% et 81%), mais des performances moindres pour *Klebsiella pneumoniae* (sensibilité de 81,1% et spécificité de 75,9%). Enfin, le temps de réponse était plus long avec les configurations *COT,* passant de 23,9 secondes pour les configurations Non *COT* (C et D) à 48,9 secondes pour les configurations *COT* (A et B).

Notre étude suggère que l’IA basée sur la technologie *COT* a un potentiel comme outil d’assistance au diagnostic en microbiologie. Ses modalités d’intégration nécessitent encore d’être précisées. Cet outil doit toujours compléter l’expertise humaine et non la remplacer.

# Research article: Prévalence et profil de résistance aux antibiotiques des bactéries hautement résistantes chez les brûlés

**Published:** 2025-11-09

**Authors:** S. Bettayeb, S. Dhraief, H. Fredj, A. Mokline, L. Thabet

**Affiliations:** 1 Laboratoire de biologie médicale, Centre de traumatologie et des grands brûlés, Université Tunis el Manar, Faculté de médecine de Tunis, UR22SP03, Tunisie; 2 Service de réanimation des brûlés, Centre de traumatologie et des grands brûlés, Tunisie

Les bactéries hautement résistantes (BHR) constituent un problème de santé publique. La résistance à plusieurs antibiotiques rend le traitement des infections causées par ces organismes, assez limité. Notre objectif de développer un programme de gestion des antibiotiques pour préserver l’efficacité des molécules est devenu crucial.

Ce travail vise à décrire la prévalence des BHR responsables d’infections chez les brûlés et de dresser leur profil de résistance aux antibiotiques dans le Centre de traumatologie et des grands brûlés (CTGB) en Tunisie.

Il s’agit d’une étude rétrospective réalisée sur une période de cinq ans de (janvier 2019 à décembre 2023) menée dans le laboratoire de biologie médicale du CTGB. Elle porte sur les BHR responsables d’infections chez les patients hospitalisés au service de réanimation des brûlés. La BHR se définit comme une bactérie présentant une résistance à au moins un agent dans toutes les classes d’antibiotiques sauf deux ou moins. Les isolats bactériens ne restent sensibles qu’à une ou deux classes.

Durant la période d’étude, 2 629 agents pathogènes ont été isolés, dont 63,3% (n=1 665) étaient des bacilles à Gram négatif (BGN), et 36,7% (n=964) des cocci à Gram positif (CGP). Les bactéries les plus fréquemment isolées étaient: *Acinetobacter baumannii* (n=342), *Klebsiella pneumoniae* (n=321) et *Pseudomonas aeruginosa* (n=229). L’étude de la prévalence annuelle des BHR de 2019 à 2023 montre une tendance significative à la hausse (r^2^: 0,903; p=0,013) avec une prévalence des BHR de 30,1% en 2019 et de 45,5% en 2023. Parmi les 2 629 souches isolées, 1 037 étaient considérées comme hautement résistantes soit une prévalence de 37,5% (n=986). Pour les BGN, 54,5% (n=908) étaient hautement résistantes. La prévalence des BHR varie en fonction de l’agent pathogène isolé. *A. baumannii* avait la prévalence de résistance la plus élevée, 89,8% (n=307), suivi par *K. pneumoniae* 76,9% (n=247) puis *P. aeruginosa* 65,5% (n=150) et *Providencia stuartii* 48,1% (n=74). Pour les CGP, 8,5% (n=78) étaient BHR, dominées par *Enteroccoccus faecium* résistant à la vancomycine (ERV) (n=42; 44,7%) et *Staphylococcus aureus* résistant à la méticilline (SARM) (n=36; 21%). Pour *A. baumannii* et *P. aeruginosa*, 66,8% et 94,7% des souches, respectivement, étaient sensibles uniquement à la colistine. La fosfomycine et la colistine étaient les associations les plus actives pour *K. pneumoniae* (36,1%). Pour *P. stuartii,* les molécules les plus actives étaient l’ertapénéme, l’aztréonam et la fosfomycine avec 29,2%, 14,8% et 41,4% de résistance respectivement. Toutes les souches de SARM et d’ERV étaient sensibles à la tigécycline et au linézolide.

La forte prévalence des BGN hautement résistantes, principalement *A. baumannii,* met en évidence la nécessité de renforcer les mesures d’hygiène et d’instaurer des protocoles de prévention des infections afin de limiter la propagation de ces bactéries hautement résistantes.

# Research article: The rising threat of multidrug-resistant infections in intensive care units: bacteriological insights from a Tunisian University Hospital

**Published:** 2025-11-09

**Authors:** Y. Maatouk, M. Marzouk, J. Mosbeh, I. Ghoufa, M. Farhat, J. Boukadida, H. Miladi, N. Hannachi

**Affiliations:** Service de microbiologie, CHU Farhat Hached de Sousse, Tunisie

Multi-drug-resistant bacteria (MDR) pose a significant threat to public health, especially in intensive care units (ICUs), where vulnerable patients are at an increased risk of developing serious infections. These bacteria often resist multiple classes of antibiotics, which complicates the treatment of healthcare-associated infections.

This study aims to analyze the bacteriological profile of MDR bacteria isolated from ICU samples at a Tunisian university hospital.

This retrospective descriptive study, conducted from 2021 to 2024, examined bacteria isolated from ICUs. The study focused on Enterobacteriaceae resistant to third-generation cephalosporins (3GC-RE), *Pseudomonas aeruginosa* resistant to ceftazidime and/or imipenem (C/IRPA), *Acinetobacter baumannii* resistant to imipenem (IRAB), and methicillin-resistant *Staphylococcus aureus* (MRSA). Antibiotic susceptibility was determined following CA-SFM/EUCAST guidelines.

A total of 527 MDR isolates were collected from the ICU over the last four years. The most prevalent isolate was IRAB (38.3%), followed by 3GC-RE (34.2%), C/IRPA (20.5%), and MRSA (7%). The main sample types were respiratory samples (57.9%), blood samples (18%), catheter samples (12%), and urine samples (7.2%). IRAB strains exhibited high resistance rates to ciprofloxacin (97%), gentamicin (96.8%), and trimethoprim-sulfamethoxazole (88.8%). Colistin and amikacin were the most effective antibiotics, with resistance rates of 3.5% and 85%, respectively. The 3GC-RE isolates were predominantly *Klebsiella pneumoniae* (83%) and showed resistance to ciprofloxacin, gentamicin, imipenem, and colistin in 88%, 55.3%, 27%, and 11.9% of cases, respectively. C/IRPA strains showed high resistance to ciprofloxacin (75%) and amikacin (62%). Most strains were susceptible to colistin, with only 4% resistant. All MRSA strains were susceptible to linezolid, tigecycline, and pristinamycin. There was a 2% resistance rate to vancomycin and a 59% resistance rate.

The emergence of MDR in ICUs is a growing concern. Due to high resistance rates, treatment options are often limited. Therefore, strengthening hospital surveillance and antibiotic stewardship programs is crucial. Rapid identification of resistant strains helps control infection outbreaks.

# Research article: État actuel de la résistance des entérobactéries au ceftazidime-avibactam au CTGB

**Published:** 2025-11-09

**Authors:** R. Ammar, S. Dhraief, MY. Fekih, L. Thabet

**Affiliations:** Laboratoire de biologie médicale, Centre de traumatologie et des grands brûlés, Université Tunis el Manar, Faculté de médecine de Tunis, UR22SP03, Tunisie

La résistance aux antibiotiques chez les entérobactéries (EB) compte parmi les principales causes de morbi-mortalité en milieu hospitalier et représente un problème majeur de santé publique.

Notre étude a pour objectif d’évaluer la résistance des entérobactéries au ceftazidime-avibactam (CZA) au Centre de traumatologie et des grands brûlés (CTGB).

Il s’agit d’une étude rétrospective descriptive menée au laboratoire de biologie médicale du CTGB sur 28 mois (septembre 2022-décembre 2024). Toutes les souches d’EB isolées à partir des prélèvements diagnostiques ont été incluses (n=3 310). L’identification bactérienne était effectuée selon les méthodes conventionnelles et l’étude de la sensibilité aux antibiotiques

selon les normes du Comité de l’antibiogramme de la Société française de microbiologie, annuellement révisées. La recherche et le typage moléculaire de carbapénèmases ont été réalisés pour un échantillon de 79 souches par une PCR GeneXpert® (Cepheid) par le kit Xpert® Carba-R détectant les gènes blaVIM, blaNDM, blalMP, blaOXA48 et blaKPC. Pour les souches résistantes à l’aztréonam (AZT) et au CZA (n=336), l’étude de l’activité de l’association CZA-AZT a été réalisée pour un échantillon de 42 souches, par deux méthodes: par ellipsométrie en superposant les bandelettes E-test CZA et AZT et par la méthode modifiée de diffusion. *Klebsiella pneumoniae* était la bactérie la plus isolée (34%) parmi les 3 310 souches d’EB colligées. Les souches d’EB étaient isolées essentiellement du service de réanimation des brûlés (36%) et du service d’anesthésie réanimation (14%). La résistance des EB au CZA était de 20,2%. Les souches étaient résistantes aux céphalosporines de 3^ème^ génération et sensibles aux carbapénèmes (C3G-R Carba-S) dans 18,8% des cas (n=622) et résistantes aux carbapénèmes (Carba-R) dans 24% des cas (n=796). Parmi les souches C3G-R Carba-S, CZA était la bêtalactamine la plus active, avec 15,3% de résistance. Pour les souches Carba-R, la résistance au CZA et à l’AZT était de 72% et 56,8%, respectivement. Une résistance à l’AZT et au CZA à la fois était observée chez 336 souches. L’étude de l’activité de l’association CZA-AZT a révélé une synergie pour l’ensemble des 42 souches testées par les deux méthodes. Pour les 79 souches typées, 8 produisaient une bêtalactamase à spectre étendu, 22 une céphalos-porinase de haut niveau, 33 étaient porteuses de blaNDM, 12 de blaOXA48, et 4 de blaNDM et de blaOXA48 à la fois. Parmi ces souches typées, 37 étaient résistantes au CZA dont 33 portant le gène blaNDM et 4 le gène bla NDM et blaOXA48 à la fois.

Les souches d’EB Carba-R étaient sensibles au CZA dans 28% des cas. Pour les souches résistantes au CZA, la sensibilité était restaurée par l’AZT montrant l’intérêt de cette association comme alternative thérapeutique pour les souches Carba-R résistantes au CZA.

# Research article: Activité *in vitro* de l’association ceftolozane/tazobactam sur les souches de *Pseudomonas aeruginosa* isolées au Centre de traumatologie et des grands brûlés (CTGB)

**Published:** 2025-11-09

**Authors:** MY. Fekih, S. Dhraief, S. Ben Aissa, L. Thabet

**Affiliations:** Laboratoire de biologie médicale, Centre de traumatologie et des grands brûlés, Université Tunis el Manar, Faculté de médecine de Tunis, UR22SP03, Tunisie

Les infections causées par *Pseudomonas aeruginosa* constituent un défi particulièrement préoccupant du fait de l’apparition de souches multirésistantes. Dans ce cadre, l’association ceftolozane/tazobactam (CT) constitue un traitement de dernier recours pour les patients atteints d’infections à *P. aeruginosa* pour lesquels le recours aux autres bêtalactamines et aux carbapénèmes n’est pas envisageable en raison des résistances.

L’objectif du travail est d’évaluer l’efficacité de l’association CT sur les souches de *P. aeruginosa* isolées au centre de traumatologie et des grands brûlés (CTGB), dans le but d’évaluer la pertinence de son introduction dans notre centre.

Il s’agit d’une étude rétrospective descriptive menée au sein du laboratoire de biologie médicale du CTGB sur une période de 41 mois (août 2021-décembre 2024). Toutes les souches de *P. aeruginosa* isolées à partir de prélèvements à visée diagnostique reçus au laboratoire ont été incluses. L’identification bactérienne a été réalisée selon les méthodes conventionnelles. L’étude de la sensibilité aux antibiotiques a été faite selon les normes du Comité de l’antibiogramme de la Société française de microbiologie, annuellement révisées.

Pendant la période d’étude, 1 205 souches non redondantes de *P. aeruginosa* ont été isolées, provenant essentiellement des services de réanimation des brûlés (51,5%) et d’anesthésieréanimation (15,6%). La résistance au CT parmi l’ensemble des souches analysées était de 29,9%, ce qui fait que cet antibiotique se classe au 3^ème^ rang des bêtalactamines les plus actives. Parmi les souches de *P. aeruginosa* isolées, 453 étaient résistantes à la ceftazidime (CAZ-R); cellesci étaient sensibles dans 27,6% des cas au CT, alors que 10,8% étaient sensibles à l’imipenème et 7,9% uniquement étaient sensibles au méropénème. Parmi les 625 souches résistantes aux carbapénèmes (CARBA-R), 42,9% étaient sensibles au CT. Parmi les souches résistantes au CT, une résistance associée à la pipéracillinetazobactam, à la gentamicine, à l’amikacine, à la ciprofloxacine et a la loslomycine a ete observee dans 99,3%, 95,1%, 78,6%, 63,8% et 43,1% des cas, respectivement. Toutes les souches de *P. aeruginosa* étaient sensibles a la colistine.

Les souches de *P. aeruginosa* isolées au CTGB se sont révélées plus sensibles a l’association CT que la plupart des autres bêtalactamines utilisées dans notre centre, confirmant qu’il s’agit d’une alternative thérapeutique prometteuse, notamment pour les souches résistantes aux autres bêtalactamines et aux carbapénèmes.

# Research article: Antibiotic resistance profiles of coagulase-negative *Staphylococcus* strains isolated from blood cultures

**Published:** 2025-11-09

**Authors:** C. Rhimi, Y. Maatouk, A. Kahloul, S. Miri, D. Rouis, J. Fathallah, H. Miladi, J. Boukadida, N. Hannachi, M. Marzouk

**Affiliations:** Microbiology laboratory, Farhat Hached university Hopsital, Sousse

Coagulase-negative staphylococci (CoNS) are part of the normal microbiota of human skin and mucous membranes. However, they are increasingly associated with bloodstream infections in hospitalized patients. The rising antibiotic resistance of CoNS, especially to methicillin, poses a significant challenge.

The aim of this study is to determine the antibiotic resistance profile of CoNS in hospitalized patients at a Tunisian university hospital.

It is a 5-year retrospective descriptive study that includes CoNS strains isolated from blood cultures. Bacteremia was diagnosed based on a combination of clinical and biological findings. Bacterial species were identified using conventional methods. Antibiotic susceptibility testing was performed according to CA-SFM/EUCAST guidelines.

During the study period, 432 CoNS strains were collected from blood cultures, primarily from the neonatology department (36.1%), pediatrics (12%), and clinical hematology (11.2%). The study population was predominantly pediatric patients (62.3%), and male (sex ratio M/F = 1.28). The most prevalent species were *Staphylococcus epidermidis* (46%; n = 199), *Staphylococcus hominis* (28.9%; n = 125), *Staphylococcus haemolyticus* (7.4%; n = 32), and *Staphylococcus xylosus* (1.4%; n = 6). A high resistance rate to methicillin was observed (73%). CoNS isolates that were resistant to methicillin showed higher resistance to other classes of antibiotics than methicillin-sensitive strains, including ciprofloxacin (67.3% *versus* 12.7%; p = 0.001), clindamycin (51% vs. 19.7%; p = 0.022), fusidic acid (57.6% vs. 15.3%; p = 0.009), norfloxacin (46% vs. 16.6%; p = 0.006), and rifampicin (64.3% vs. 19.8%; p < 0.001). The most effective antibiotics were vancomycin, pristinamycin, linezolid, and tigecycline, with resistance rates of 4%, 0.4%, 3%, and 0%, respectively.

This study emphasizes the significant prevalence of methicillin-resistant CoNS in blood cultures and their increased resistance to other antibiotics.

# Research article: Évolution de l’antibiorésistance des bacilles à Gram négatif non fermentaires au CHU de Monastir

**Published:** 2025-11-09

**Authors:** E. Mhamdi, A. Abassi, H. Rhim, B. Kouidhi, M. Bhouri, O. Haddad, Y. Kadri, M. Mastouri

**Affiliations:** Laboratoire de microbiologie, CHU Fattouma Bourguiba, Monastir, Tunisie

Les bacilles à Gram négatif non fermentaires (BGNNF) sont des pathogènes opportunistes responsables d’infections nosocomiales sévères. Ils ont une capacité à développer des multirésistances à différentes classes d’antibiotiques ce qui pose un défi thérapeutique majeur.

Cette étude vise à évaluer l’évolution des résistances aux antibiotiques de ces BGNNF au CHU de Monastir.

Il s’agit d’une étude rétrospective sur 5 ans (20202024), ayant porté sur toutes les souches de *Pseudomonas aeruginosa, Acinetobacter baumannii* et *Stenotrophomonas maltophilia* isolées au laboratoire de microbiologie du CHU de Monastir. L’identification bactérienne a été effectuée selon les méthodes conventionnelles et la sensibilité aux antibiotiques a été interprétée selon les recommandations du Comité de l’antibiogramme de la Société française de microbiologie en vigueur. Un total de 2 703 souches de BGNNF a été isolé dont 68,5% ont été des isolats de *P. aeruginosa,* suivi par *A. baumannii* (25,6%) et *S. maltophilia* (5,8%). Durant la période d’étude, *P. aeruginosa* résistant à la ceftazidime (PARC) et/ou à l’imipénème (PARI) a été isolé dans 15,3% et 17,1% respectivement sans tendance nette tandis que *A. baumannii* résistant à l’imipénème (ABRI) a été retrouvé dans 89,5% montrant une tendance à la hausse, passant de 84,9% en 2020 à 88,7% en 2024, atteignant un pic de 93,8% en 2021. La résistance aux fluoroquinolones a varié entre 19,5% et 30,5% avec un pic en 2023 pour *P. aeruginosa*. Elle est restée élevée, autour de 91-93% pour *A. baumannii.* Concernant les aminosides, la résistance est passée de 22,6% à 10,4% pour *P. aeruginosa* et de de 70,9% à 89,2% pour *A. baumannii.* La colistine est restée active, avec 4,6% et 11,8% pour *P. aeruginosa* et *A. baumannii* respectivement. S. *maltophilia* avait une résistance à la ceftazidime stable, autour de 57,6%. La lévofloxacine a montré une augmentation modérée de la résistance, atteignant 8,2% en 2024 tandis que le cotrimoxazole a vu une hausse importante de résistance, passant de 2,7% à 22,4%.

Cette étude révèle une augmentation préoccupante de la résistance notamment aux carbapénèmes et à la colistine chez *A. baumannii.* Une surveillance renforcée et une optimisation des stratégies d’antibiothérapie sont essentielles pour limiter la propagation des souches multirésistantes en milieu hospitalier.

# Research article: Performance des méthodes phénotypiques dans le dépistage de la résistance aux bêtalactamines et des mutations ftsI chez *Haemophilus influenzae*

**Published:** 2025-11-09

**Authors:** Y. Chelbi, K. Meftah, AE. Deghmane, S. Mhimdil, F. Aloui, A. Bouafsoun, E. Hong, MK. Taha, H. Smaoui

**Affiliations:** 1 Laboratoire de microbiologie, Hôpital d’enfants Béchir Hamza de Tunis, LR18ES39, Faculté de médecine de Tunis, Université Tunis el Manar, Tunisie; 2 Unité des infections bactériennes invasives, Centre national de référence des méningocoques et *Haemophilus influenzae,* Institut Pasteur Paris, France

La résistance *dHaemophilus influenzae (Hi)* aux bêtalactamines est devenue préoccupante ces dernières années. Le Comité européen pour les tests de sensibilité aux antimicrobiens (EUCAST) recommande d’utiliser le disque de benzylpénicilline 1pg (PG1) pour dépister la résistance aux bêtalactamines.

L’objectif de cette étude était d’évaluer les performances de la PG1 dans le dépistage des mutations ftsI et de la résistance aux bêtalactamines chez les souches invasives de *Hi.*

Une étude rétrospective a été menée au laboratoire de microbiologie de l’Hôpital d’enfants Béchir Hamza de Tunis (mai 2013-avril 2023). Les souches de Hi isolées dans des sites stériles ont été incluses. Un disque PG1 a été testé selon les recommandations de l’EUCAST. Les concentrations minimales inhibitrices de l’ampicilline (AMP), amoxicilline acide-clavulanique (AMC) et du céfotaxime (CTX) ont été déterminées par bandelette E-test (Biomérieux^®^). La détection des gènes de bêta-lactamases, bla-TEM1 et bla-ROB1,

et des mutations du gène ftsI a été réalisée par séquençage du génome complet (Illumina^®^). Quarante-cinq souches de *Hi* ont été colligées. Le dépistage de la résistance aux bêtalactamines par la PG1 était positif chez 25 souches (56%). La résistance à l’AMP, l’AMC et au CTX était de 19/45 (42%), 9/45 (20%) et 2/45 (4%) respectivement. Toutes les souches résistantes à l’AMP avaient un dépistage par la PG1 positif. Cependant, six souches étaient sensibles avec un dépistage positif et portaient des mutations critiques conférant une résistance à l’AMP (n=2) et/ou au CTX (n=4) (kappa=0,73; p<0,0001). Quatorze souches (31%) portaient le gène bla-TEMl. Les mutations critiques ftsI ont été détectées chez 21/45 (47%) des souches et chez 17/31 (55%) des souches bla-TEM1 négatives. Dans ce dernier groupe, 5/17 des souches portant des mutations ftsI n’ont pas été détectées par la PG1 (kappa=0,68; p<0,0001). La sensibilité, la spécificité, la valeur prédictive négative et positive du dépistage par la PG1 des mutations ftsI étaient de 71%, 100%, 74% et 100% respectivement.

Les méthodes phénotypiques ont montré d’excellents résultats dans le dépistage des souches résistantes aux bêtalactamines. Cependant, la positivité du dépistage par la PG devrait être interprétée avec précaution. Ces méthodes ont présenté une excellente spécificité pour la détection des mutations ftsI, mais leur sensibilité reste limitée.

# Research article: Évaluation de l’impact de l’introduction du Biofire^®^ Film Array Pneumonia dans une unité de réanimation

**Published:** 2025-11-09

**Authors:** S. Saidani, A. Jebri, M. Harrabi, M. Rahoui, A. Assily, L. Kanzari, A. Ferjani, L. Amdouni, F. Sellami, I. Boutiba

**Affiliations:** Service d’anesthésie et de réanimation, Hôpital Charles Nicolle, Tunis, Tunisie

Les pneumopathies acquises sous ventilation mécanique (PAVM) sont fréquentes particulièrement en réanimation. Une antibiothérapie probabiliste souvent à large spectre adaptée à l’écologie du service. Le panel Biofire® Film Array pneumonía a été introduit pour le diagnostic rapide des PAVM.

L’objectif de notre travail était d’évaluer l’effet de l’impact de l’introduction du panel Biofire^®^ Film Array pneumonia sur la prescription de l’antibiothérapie et sur le pronostic des patients. Il s’agit d’une étude rétrospective, menée au service d’anesthésie réanimation sur une période de 5 mois, d’octobre 2024 à février 2025. Nous avons inclus les patients présentant une PAVM. Une étude microbiologique conventionnelle et une analyse par PCR multiplex par le panel Biofire^®^ Film Array pneumonia ont été effectuées. Nous avons étudié l’adéquation entre les résultats de la PCR multiplex et la microbiologie et son impact sur l’adaptation de l’antibiothérapie. Nous avons par ailleurs comparé la consommation globale d’antibiothérapie à large spectre et la déclaration de bactéries multirésistantes (BMR) lors de la même période une année auparavant.

Nous avons recueilli 30 patients ayant présenté une PAVM dont quatre ont été exclus. Nous avons donc analysé 26 patients. L’âge moyen était 55±18 ans et une nette prédominance masculine était notée (24 hommes). Le motif d’amission était une détresse respiratoire dans 35% des cas et un traumatisme crânien grave dans 31% des cas. Le score SOFA était 7±3 à l’admission. Les patients ont eu une ventilation invasive dans 88% des cas. Le délai médian entre prélèvement et résultat de la PCR multiplex était de 2 h (1,75 - 10,5) et de 48 h (48 - 72) pour la microbiologie conventionnelle. Une introduction ou une modification de l’antibiothérapie était faite pour 11 patients après le résultat de la PCR multiplex et dans 4 cas après les résultats de la bactériologie standard. La concordance entre les résultats de la multiplex et de la bactériologie standard était de 50%. L’évolution a été marquée par un décès dans 57% des cas. La consommation d’antibiotiques était plus basse après introduction de la PCR multiplex: 2 025 doses d’octobre 2023 à février 2025 *versus* 1 402 doses d’octobre 2024 à février 2025. Nous n’avons pas noté de changement significatif pour le nombre de BMR déclarés (48 entre 2023 et 2024 et 50 entre 2024 et 2025) mais nous avons noté moins de carbapénémases.

L’introduction du panel Biofire^®^ Film Array pneumonia a permis une adaptation plus rapide de l’antibiothérapie et une moindre consommation d’antibiotiques. Ces résultats prometteurs sont à valider par une étude prospective à plus large échelle.

# Research article: Bactériémies communautaires à l’Hôpital Charles Nicolle (20202024): profil bactériologique et sensibilité aux antibiotiques

**Published:** 2025-11-09

**Authors:** S. Sfaxi, L. Kanzari1, A. Ben Massoud, A. Rehaiem, A. Fakhfekh, S. Ferjani, A. Ferjani, I. Boutiba Ben Boubaker

**Affiliations:** 1 Laboratoire de microbiologie, Hôpital Charles Nicolle, Tunis, Tunisie; 2 Université Tunis El Manar, Faculté de médecine de Tunis, Laboratoire de recherche résistance aux antimicrobiens « LR99ES09 », Tunis, Tunisie

Les bactériémies communautaires représentent un défi majeur en infectiologie, en raison de leur impact sur la morbidité et la mortalité des patients, exacerbées par l’augmentation des bactéries multirésistantes (BMR).

Cette étude a pour objectif d’évaluer le profil bactériologique et la sensibilité aux antibiotiques des bactéries incriminées dans les bactériémies communautaires à l’Hôpital Charles Nicolle.

Il s’agit d’une étude rétrospective menée entre 2020 et 2024 au laboratoire de microbiologie. Tous les cas de bactériémies communautaires confirmés par hémoculture chez les patients hospitalisés ont été inclus. Une bactériémie était considérée comme communautaire si les hémocultures étaient prélevées dans les 48 heures suivant l’admission chez un patient présentant des signes d’infection à l’admission. L’identification bactérienne a été réalisée selon des méthodes conventionnelles et la sensibilité aux antibiotiques a été évaluée conformément aux recommandations du Comité de l’antibiogramme de la Société française de microbiologie.

Au total, 932 souches bactériennes d’origine communautaire ont été isolées, réparties en 460 cocci Gram positif et 472 bacilles Gram négatif. Les espèces bactériennes les plus fréquemment identifiées étaient *Staphylococcus aureus* (26%), *Escherichia coli* (17,6%) et *Klebsiella pneumoniae* (15%). Elles provenaient essentiellement du service des urgences (17,4%), majoritairement *S. aureus* (54,9%) et du service de néonatologie (17,1%), majoritairement *K. pneumoniae* (17,6%). Les souches de *S. aureus* étaient résistantes à la méticilline dans 12,4% des cas, à la gentamicine dans 2,9% et à la ciprofloxacine dans 11,6%. Elles étaient par ailleurs sensibles aux glycopeptides. *E. coli* était résistant aux céphalosporines de 3^e^ génération dans 21,3% des cas, à l’amikacine dans 5,5% et à la ciprofloxacine dans 30,5%. Aucune résistance à l’imipenème n’a été retrouvée. Au total, 162 isolats (17,4%) étaient classés comme BMR, comprenant 86 entérobactéries productrices de β-lactamases à spectre élargi, 15 souches productrices de carbapénémases, 9 *P. aeruginosa* et 22 *A. baumannii* résistants à l’imipenème.

L’isolement de BMR responsables de bactériémies communautaires dans notre hôpital souligne la propagation de la résistance aux antibiotiques en dehors des milieux hospitaliers, posant un défi de santé publique global.

# Research article: Antibiothérapie locale par ciment imprégné: une stratégie optimisée pour le traitement des infections osseuses chroniques

**Published:** 2025-11-09

**Authors:** S. Hmila, R. Ayari, H. Chahed, K. Khelil, Y. Mallat, T. Znagui

**Affiliations:** Service d’orthopédie et traumatologie. Hôpital militaire principal d’instruction de Tunis, Tunisie

Les infections osseuses chroniques posent un défi thérapeutique en raison de la mauvaise vascularisation des tissus infectés induisant une faible diffusion des antibiotiques systémiques. Le ciment imprégné par des antibiotiques permet une diffusion ciblée permettant l’éradication de l’infection.

L’objectif était d’évaluer l’efficacité de l’antibiothérapie locale par ciment imprégné par des antibiotiques dans le traitement des ostéites chroniques post-traumatiques, en analysant le taux d’éradication infectieuse, la consolidation osseuse et les complications associées.

Nous avons mené une étude rétrospective incluant les patients traités dans le service d’orthopédie et traumatologie de l’Hôpital militaire principal d’instruction de Tunis pour une ostéite chronique du tibia ou du calcanéum entre 2012 et 2022. Les patients présentaient soit une ostéite du calcanéum suite à un pied de mine soit une ostéite du tibia après fracture ouverte. Tous ont bénéficié d’un débridement chirurgical extensif suivi d’une cimentoplastie imprégnée par des antibiotiques adaptés. L’évaluation post-opératoire incluait la disparition des signes infectieux, le délai de cicatrisation et la récupération fonctionnelle. Les complications locales et systémiques ont été analysées avec un recul moyen de 24 mois.

Cinquante patients ont été inclus, 46 (92%) ont présenté une éradication complète de l’infection sans récidive après un suivi moyen de 24 mois. La cicatrisation a été obtenue en moyenne après 4 semaines et la consolidation après 3 mois. Une amélioration fonctionnelle significative a été constatée dès la 6^e^ semaine post-opératoire, permettant à la majorité des patients de reprendre un appui partiel progressif. Dans le groupe des ostéites calcanéennes (n=40), 37 patients (92,5%) ont guéri sans récidive tandis que 3 ont nécessité une réintervention pour écoulement persistant, avec une évolution favorable après soins locaux et antibiothérapie adaptée. Parmi les 10 patients ayant une ostéite tibiale, 8 ont guéri sans récidive. Un patient a nécessité une prise en charge complémentaire incluant une greffe osseuse et un patient a présenté un réveil septique avec pandiaphysite indiquant une amputation. Trois patients (6%) ont présenté des complications locales mineures, incluant des réactions inflammatoires au ciment, traitées par soins locaux. Aucun effet indésirable systémique lié à la diffusion des antibiotiques locaux n’a été observé. La cimentoplastie imprégnée par des antibiotiques représente une stratégie efficace pour traiter les ostéites chroniques, avec une morbidité réduite et un taux de guérison satisfaisant. Des études prospectives randomisées restent nécessaires pour valider ces résultats et affiner les protocoles thérapeutiques.

# Research article: *Enterobacter cloacae*: épidémiologie et antibiorésistance sur 12 ans au CHU Fattouma Bourguiba de Monastir (2012-2023)

**Published:** 2025-11-09

**Authors:** S. Chemli, N. Daghari, N. Sayeh, Y. Kadri, H. Rhim, O. Hadded, M. Mastouri

**Affiliations:** Laboratoire de microbiologie, CHU Fattouma Bourguiba, Monastir, Tunisie

*Enterobacter cloacae* est la 3^e^ entérobactérie impliquée dans des infections variées chez l’Homme. Cette espèce bactérienne peut présenter une impasse thérapeutique du fait de sa capacité prodigieuse de développer des résistances aux antibiotiques.

L’objectif de notre travail est d’étudier le profil épidémiologique des souches d’E. *cloacae* et d’établir un état des lieux sur son antibiorésistance par une étude monocentrique rétrospective descriptive menée au laboratoire de microbiologie au sein du CHU Fattouma Bourguiba de Monastir sur 12 ans (1^er^ janvier 2012-31 décembre 2023). Toutes les souches non redondantes d’E. *cloacae* issues de prélèvements à visée diagnostique ont été incluses. L’identification bactérienne a été effectuée par les méthodes conventionnelles. L’étude de la sensibilité aux antibiotiques s’est basée sur les recommandations actualisées du Comité de l’antibiogramme de la Société française de microbiologie.

Ont été colligées 1 669 souches d’*E. cloacae* avec une prévalence de 3,5%. Ces isolats sont parvenus majoritairement de sujets adultes (60%). Le sex-ratio (H/F) a été de 2,6. Les souches ont émané principalement de services chirurgicaux (n= 699,41,8%). Les unités de réanimation ont hébergé 296 souches correspondant à 17,7% des souches. Ces isolats ont essentiellement émané de prélèvements de pus (n=706,42,3%) ou des urines (n= 467,28%). La cinétique des résistances aux antibiotiques a été relativement stable en dehors des céphalosporines de 3^ème^ génération (C3G) et des carbapénèmes. Les résistances envers ces molécules ont augmenté significativement à partir de l’année 2021. Plus d’un quart des souches était constitué de bactéries multirésistantes (BMR) montrant un taux de résistance aux C3G de 26,2%. L’activité bactéricide des carbapénèmes a été inhibée dans 6,2% des cas. Ces BMR ont été trouvées dans la majorité des cas dans des services hospitaliers: la chirurgie générale (30%) et réanimation chirurgicale (21%). Les souches isolées par uroculture ont représenté 40% des souches résistantes aux C3G et 48% des souches résistantes aux carbapénèmases provenaient des prélèvements de pus.

La prévalence d’E. *cloacae* tend à augmenter de plus en plus. Les chiffres de résistance aux C3G et aux carbapénèmes sont considérables. L’émergence de souches BMR nécessite des efforts plus importants pour limiter leur dissémination.

# Research article: La spondylodiscite infectieuse: à propos de 131 cas

**Published:** 2025-11-09

**Authors:** R. Bougossa, O. Jomaa, E. Khouaja, S. Baccouche, M. Jguirim, I. Bejia, M. Younes, F. Larbi

**Affiliations:** 1 Service d’endocrinologie et de médecine interne, CHU Taher Sfar, Mahdia, Tunisie; 2 Service de rhumatologie, CHU Taher Sfar, Mahdia, Tunisie; 3 Service de rhumatologie, CHU Fattouma Bourguiba, Monastir, Tunisie

La spondylodiscite infectieuse (SPDI) est une infection rare mais grave qui peut mettre en jeu le pronostic vital et fonctionnel.

L’objectif était de décrire les caractéristiques épidémio-cliniques, paracliniques, thérapeutiques et évolutives des SPDI grâce à une étude descriptive rétrospective (2012-2024) incluant tous les patients hospitalisés au CHU Taher Sfar de Mahdia en Tunisie pour une SPDI documentée ou présumée.

Au total, 131 patients (67 hommes et 64 femmes) étaient colligés dans notre étude, âgés en moyenne de 57,7±15,7 ans. La comorbidité la plus fréquemment associée était le diabète (n=47; 35,9%). Une consommation de produits laitiers non pasteurisés et un contage tuberculeux étaient notés dans 19,8% et 6,1% des cas respectivement. Les signes cliniques étaient dominés par les rachialgies (96,9%), les radiculalgies (40,5%) et la fièvre (48,1%). Un déficit moteur était observé dans 7 cas (5,3%). Le taux médian de la protéine C-réactive était de 44 mg/l. À l’IRM rachidienne, les érosions des plateaux vertébraux, une épidurite et les abcès des parties molles paravertébrales étaient observés dans 70,2%, 64,9% et 58% des cas respectivement. Les hémocultures étaient positives dans 22 cas (16,8%). Une ponction-biopsie discovertébrale était pratiquée dans 65 cas (49,6%). L’intradermoréaction à la tuberculine et la sérologie de Wright étaient positives dans 13 cas (9,9%) et 16 cas (12,2%) respectivement. Les cas de SPDI étaient répartis en 86 cas (65,6%) de spondylodiscites à germes pyogènes (SPDP), 28 cas (21,4%) de spondylodiscite tuberculeuse et 17 cas (13,0%) de spondylodiscite brucellienne. Pour les SPDP, *Staphylococcus aureus* était le germe le plus fréquemment identifié (62,8%). Une antibiothérapie adaptée était prescrite dans tous les cas et un drainage des abcès paravertébraux était préconisé dans 12 cas (9,2%). L’évolution était favorable dans 87% des cas et 11 patients (8,4%) présentaient des séquelles motrices et/ou douloureuses.

Une bonne enquête étiologique précoce et une prise en charge adéquate permettraient d’améliorer le pronostic des SPDI.

# Research article: Profil cytokinique des souspopulations de cellules T dans le liquide cérébro-spinal de patients atteints d’encéphalite virale

**Published:** 2025-11-09

**Authors:** K. Tebibi, R. Ben Laamari, Z. Saied, O. Maghrebi, H. Touzi, Z. Meddeb, S. Ben Sassi, H. Triki, M. Belghith, D. Rezig

**Affiliations:** 1 Laboratoire de recherche “Virus, Vectors and Hosts: One Health Approach and Technological Innovation for a Better Health”, LR20IPT02, Institut Pasteur de Tunis, Tunisie; 2 Faculté des sciences de Tunis, Université Tunis El Manar, Campus universitaire, El Manar, Tunis 2092, Tunisie; 3 Laboratoire de transmission, contrôle, et immunobiologie des infections, Institut Pasteur de Tunis, Université Tunis El Manar, Tunisie; 4 Service de neurologie, Institut Mongi Ben Hmida, Tunis, Tunisie; 5 Faculté de médecine de Tunis, Université Tunis El Manar, Tunisie; 6 Laboratoire de virologie clinique, Laboratoire de référence OMS pour la poliomyélite et la rougeole, Institut Pasteur de Tunis, Université Tunis El Manar, Tunis 1002, Tunisie

L’encéphalite virale est une inflammation du parenchyme cérébral résultant d’une invasion virale directe. Le diagnostic exige principalement l’analyse du liquide cérébro-spinal (LCS). Malgré le développement des techniques de biologie moléculaire, l’étiologie des encéphalites reste inconnue dans la majorité des cas. L’objectif était la recherche de virus associés, l’étude du profil cytokinique des sous-populations de cellules T dans le LCS de patients atteints d’encéphalite virale et la comparaison des profils obtenus avec un groupe témoin.

Au total, 15 LCS issus de patients atteints d’encéphalite virale et recrutés à l’Institut national de neurologie Mongi Ben Hamida ont été analysés. Le groupe témoin comprenait 22 individus présentant des migraines persistantes ayant nécessité une ponction lombaire sans étiologie infectieuse retrouvée. Des PCR multiplex et qRT-PCR ont été utilisées pour rechercher les virus les plus souvent incriminés dans les encéphalites virales. Des PCR quantitatives en temps réel moyennant SYBR Green ont été utilisées pour évaluer l’expression de certaines cytokines (IFN-y, IL-1b, TNF-a, IL-17, IL-6, IL-4, and IL-10) et de facteurs de transcription (T-bet, GATA-3, RoR-γt, and FOXP3) dans les LCS.

Profil virologique: les membres de la famille des Herpesviridae sont les virus les plus fréquemment observés chez les patients atteints d’encéphalite virale. Parmi les 15 patients étudiés, 8 cas d’infection à cytomégalovirus, 4 cas d’infection par le virus varicelle-zona, 1 cas de rougeole, une co-infection virus varicelle-zona (VZV)/Epstein Barr et un cas d’infection par entérovirus ont été retrouvés. Aucun cas d’infection par les virus West Nile ou Toscana n’a été identifié. Comparaison des profils immunologiques: nous n’avons pas trouvé de différence significative dans la moyenne des facteurs de transcription (T-bet, GATA-3, RoR-γt, et FOXP3) ainsi que des cytokines (IFN-c, IL-4 et IL-10) étudiés entre les deux groupes. De manière intéressante, nous avons remarqué une diminution significative du niveau d’ARNm de l’IL-17 en cas d’encéphalite virale. Nous avons également évalué la balance Th1/Th2 et nos résultats ont indiqué une augmentation de la réponse Th1 avec une expression élevée de T-bet ainsi qu’un déséquilibre du ratio Th17/Treg, caractérisé par une diminution de l’expression des Treg chez les patients avec une encéphalite virale. Finalement, nous avons noté une augmentation significative de l’expression de l’IL-6 et une baisse de l’expression des TNF-a and IL-1b chez les patients avec une encéphalite virale par rapport au groupe témoin.

En conclusion, différents virus ont été retrouvés chez les cas d’encéphalite. Une prédominance Th1 et une modification de l’équilibre entre composantes régulatrices et pro-inflammatoires ont été notées. D’autres recherches sont nécessaires pour découvrir les mécanismes des changements immunologiques et leurs implications cliniques.

# Research article: Altered n-3 fatty acids metabolism is associated with mortality in severe Covid-19

**Published:** 2025-11-09

**Authors:** A. Bouabdallah, R. Abdelmalek, MB. Hammami, MK. Ben Fradj, L. Amman, M. Feki

**Affiliations:** 1 Laboratory of Biochemistry, LR99ES11, Rabta Hospital of Tunis, Faculty of Medicine of Tunis, Tunisia; 2 Infectious diseases Department, Rabta Hospital of Tunis, Faculty of Medicine of Tunis, Tunisia

Omega-3 polyunsaturated fatty acids (PUFAs) have anti-inflammatory properties, acting both directly and indirectly through their pro-resolving derivatives. These fatty acids are essential for resolving inflammation. The human body cannot synthesize alpha-linolenic acid (ALA) and must obtain it through diet. Eicosapentaenoic acid (EPA), docosapentaenoic acid (DPA), and docosahexaenoic acid (DHA) are obtained from seafood or synthesized from ALA.

This study examined the association between plasma n-3 PUFAs and mortality in patients with severe cases of the disease.

We conducted a prospective study involving patients hospitalized with severe cases of the disease. The patients received standard treatment and were monitored until discharge or death. Plasma free n-3 PUFAs were measured using an LC-MS/MS method. We applied a general linear model, adjusting for age, and performed ROC analyses to test the association between n-3 PUFAs and mortality.

Of the 134 patients, 21 died during their hospital stay. There were no significant differences in smoking or comorbidities between the deceased and surviving patients. However, the former group was older (72.1 ± 10.9 vs. 61.7 ± 13.9 years; p < 0.001). Plasma ALA levels were higher (median [interquartile range (IQR)] 1.47 [1.33] vs. 0.91 [0.94] pg/mL, p = 0.005), while plasma EPA levels were lower (0.12 [0.15] vs. 0.15 [0.38] pg/ mL, p = 0. Deceased patients had lower levels of DPA (0.19 [0.19] vs. 0.36 [0.31] pg/mL, p = 0.008) and DHA (0.56 [0.58] vs. 0.82 [0.974] μg/mL, p = 0.087). The ALA/(EPA + DPA + DHA) ratio was substantially higher in deceased patients (1.59 [1.42] vs. 0.58 [0.40]). A ratio of 0.93 predicts death with 72% sensitivity and 80% specificity (area under the curve [AUC], 0.77 [0.62-0.92]). An increased ALA/(EPA + DPA + DHA) ratio indicates decreased endogenous synthesis, high oxidation/peroxidation of n-3 PUFAs, or low seafood intake. A high ratio indicates altered PUFA metabolism and predicts death in severe cases of Covid-19 with high specificity. Due to the anti-inflammatory and beneficial properties of n-3 PUFAs, a deficit of these nutrients may contribute to inflammation and worsen outcomes for patients with severe cases of the disease. A balanced diet or supplementation that ensures adequate n-3 PUFA status could prevent poor outcomes in patients with severe cases of the disease and other infectious and inflammatory diseases.

# Research article: SARS-CoV-2 Infection and genetic evolution in patients with primary immunodeficiency

**Published:** 2025-11-09

**Authors:** H. Khemiri, I. Ben Salem, I. Ben Fraj, A. Lorusso, N. Mekki, I. Mangone, M. Gdoura, A. Di Pasqual, C. Cammà, V. Di Lollo, A. Cherni, H. Touzi, A. Sadraoui, Z. Meddeb, N. Hogga, I. Ben Mustapha, MR. Barbouche, M. Ouederni, H. Triki, S. Haddad-Boubaker

**Affiliations:** 1 Laboratory of Clinical Virology, WHO Regional Reference Laboratory for Poliomyelitis and Measles for in the Eastern Mediterranean Region, Institut Pasteur de Tunis, University of Tunis El Manar, Tunis, Tunisia; 2 Research Laboratory “Viruses, Vectors and Hosts” (LR20IPT02), Institut Pasteur de Tunis, University of Tunis El Manar, Tunis, Tunisia; 3 Pediatric Department of the National Center of Bone Marrow Transplantation, Tunis, Tunisia; 4 Istituto Zooprofilattico Sperimentale dell’Abruzzo e del Molise, Teramo, Italy; 5 Laboratory of Transmission, Control and Immunobiology of Infection (LR 11 IPT 02), Institut Pasteur de Tunis, University of Tunis El Manar, Tunis, Tunisia; 6 Faculty of Medicine, University of Tunis El Manar, Tunis, Tunisia; 7 Department of Microbiology, Immunology and Infectious Diseases, College of Medicine and Health Sciences, Arabian Gulf University, Manama, Bahrain Patients with primary immunodeficiencies (PIDs) are vulnerable and at increased risk for severe cases and prolonged SARS-CoV-2 infections. Persistent SARS-CoV-2 can contribute to community transmission and lead to the emergence of new viral strains. This study investigates SARS-CoV-2 infection and genetic evolution in pediatric patients with PID.

Samples were collected from five pediatric patients with PID, including 15 nasopharyngeal specimens and 10 stool specimens. RNA detection was performed using RT-qPCR targeting the RdRp and N genes. Whole genome sequencing was conducted using the Illumina SARS-CoV-2 (CovideSeq) protocol on a NexSeq 1000 sequencer. Data analysis was performed using the Nextflow nf-core/viralrecon pipeline. Variant assignment was performed using Pangolin software (version 4.3). Phylodynamic analysis was performed using BEAST software.

Viral RNA was detected in nasopharyngeal samples for periods ranging from seven to 28 days; however, only one patient excreted RNA in stool samples for up to 28 days. Patients with antibody deficiencies and combined immune deficiencies exhibited the longest periods of viral shedding. One patient developed severe post-COVID-19 complications and died. The sequences obtained from the excreted strains were identified as Delta (AY.122) and Omicron (BA.1.1). A phylodynamic analysis was constructed using the strains obtained from the patient who had a fatal outcome and prolonged excretion. The analysis revealed a close genetic relationship between sequences obtained on days 1, 7, 14, and 21. However, the sequence obtained on day 28 exhibited considerable genetic distance from the preceding sequences.

Our findings underscore the prolonged excretion of SARS-CoV-2 RNA in patients with antibody and combined immune deficiencies. This emphasizes the need for long-term surveillance of viral evolution in patients with primary immunodeficiency.

# Research article: Diversity and global distribution of recombinant SARS-CoV-2 subvariants: insights into hotspot locations and evolutionary dynamics

**Published:** 2025-11-09

**Authors:** M. Khedhiri, S. Haddad-Boubaker, M. Arbi, K. Ayouni, O. Souiai, S. Dhouib, N. Ghanmi, A. Benkahla, H. Triki

**Affiliations:** Laboratoire de virologie clinique, Institut Pasteur de Tunis, Tunisie

Severe acute respiratory syndrome coronavirus 2 (SARS-CoV-2) has significantly impacted global health. Recombination plays a critical role in the evolution of RNA viruses, leading to the emergence of novel, epidemic-prone variants.

This study analysed the diversity and geographic distribution of recombinant SARS-CoV-2 Omicron subvariants.

From July 2022 to January 2023, 44,230 complete SARS-CoV-2 genome sequences were obtained from the GISAID and NCBI databases. Of these, 43,749 were Omicron BQ subvariants and 484 were ML-designated. The sequences were filtered to exclude those with more than 10% ambiguous nucleotides. Two recombination events were identified using the MAFFT and 3seq programs. Statistical analyses of geographic distribution, recombinant type frequency, and recombinant region frequency were performed using R packages.

BQ recombinant sequences accounted for 56.91% of the total, while ML recombinant sequences accounted for 82.20%. Most BQ recombinant variants (>80%) underwent recombination with XBW, XBC.1, and Wuhan-Hu-1, while other variants exhibited low recombination rates (<20%). ML subvariants were double recombinants with XBB.1.5/XBB.2 (53.61%) and XBB.1.5/XBB.1.2 (56.39%). BQ recombinant regions were primarily located in the 3’UTR (25.87%), spike (23.24%), and matrix (18.78%) regions of the genome. Most

ML subvariants showed recombination in the NSP6/NSP7 and NSP9/NSP10 regions. Recombinant strains primarily circulated in the Americas, Europe, and Asia. The United States had the highest detection rate (94.98%), while the United Kingdom (3.06%) and Australia (0.63%) had lower rates.

This study highlights the widespread distribution and diversity of recombinant SARS-CoV-2 subvariants, as well as their distinct geographic patterns. These results underscore the role of recombination in shaping viral evolution and emphasize the necessity of continuous genomic surveillance to track emerging recombinant variants.

# Research article: Les Rhinovirus/Enterovirus, VRS et virus grippaux: quel impact de la pandémie de Covid-19 sur leur circulation en Tunisie?

**Published:** 2025-11-09

**Authors:** F. Smaoui, A. Taktak, S. Gargouri, A. Chtourou, R. Kharrat, A. Rebai, L. Feki-Berrajah, H. Karray-Hakim

**Affiliations:** 1 Laboratoire de microbiologie, CHU Habib Bourguiba, Sfax, Tunisie; 2 Centre de biotechnologie de Sfax, Tunisie

La pandémie de Covid-19 a été marquée par d’importantes mesures de santé publique visant à limiter la transmission du SARS-CoV-2. Toutefois, leur impact sur l’épidémiologie des autres virus respiratoires reste à investiguer.

Notre objectif était d’évaluer l’impact de la pandémie de Covid-19 sur l’épidémiologie des rhinovirus et entérovirus (RV/EV), du virus respiratoire syncytial (VRS) et des virus grippaux en Tunisie.

Cette étude a porté sur 1 326 échantillons nasopharyngés collectés entre novembre 2019 et février 2023 au laboratoire de microbiologie du CHU Habib Bourguiba-Sfax. L’identification des RV/EV, du VRS et des virus grippaux a été réalisée par RT-PCR. Les taux de détection ont été comparés aux tendances de positivité du SARS-CoV-2 afin d’évaluer l’impact des vagues pandémiques sur la circulation de ces virus. L’étude statistique a été réalisée par le logiciel RStudio. Avant la pandémie, les RV/EVs, le VRS et les virus grippaux ont co-circulé durant l’hiver 2019/2020 avec des taux de positivité de 25%, 10% et 9%, respectivement. Pendant la pandémie, les RV/EV sont restés largement prévalents tout au long de la période étudiée, contrairement au VRS et aux virus grippaux, dont la circulation a fortement diminué, atteignant des taux inférieurs à 1,5% et 1% entre octobre 2020 et août 2021. L’analyse statistique a montré l’absence de variation significative du taux de positivité des RV/EV lorsque la prévalence du SARS-CoV-2 dépassait 30% (p=0,508), bien qu’une tendance à la baisse ait été relevée lors des vagues épidémiques les plus marquées (>35%, p=0,076). En revanche, la circulation du VRS et des virus grippaux a été significativement réduite lorsque la prévalence du SARS-CoV-2 dépassait 30% (p<0,001 et p=0,014, respectivement).

La baisse de la prévalence des VRS et des virus grippaux pourrait être attribuée aux interventions de santé publique et à l’éventuelle interférence virale avec le SARS-CoV-2. En revanche, la circulation persistante des RV/EV suggère une plus grande résistance aux mesures sanitaires.

# Research article: Genetic variability of hepatitis A virus after vaccine introduction to the national vaccination program

**Published:** 2025-11-09

**Authors:** K. Ayouni, A. Chouikha, A. Jaziri, H. Touzi, W. Hammemi, H. Triki

**Affiliations:** Laboratoire de virologie clinique, Institut Pasteur de Tunis, Tunisie

Severe hepatitis A outbreaks in Tunisia led to the inclusion of the hepatitis A vaccine in the national immunization schedule. Understanding the molecular epidemiology of circulating strains after vaccination is essential for evaluating the vaccine’s effectiveness and improving disease control strategies.

The goal of this study was to update the genetic diversity of hepatitis A virus (HAV) strains in Tunisia after the hepatitis A vaccine was introduced into the national immunization schedule. Thirty-one IgM anti-HAV-positive samples collected during diagnostic activities at the Pasteur Institute of Tunis’s Laboratory of Clinical Virology from 2019 to 2022 were examined. Viral RNA was extracted using the QIAmp Viral RNA Mini Kit. We detected viral RNA through real-time RT-PCR. Positive samples were selected for genotyping. We amplified a 394 bp region in the VP1/2A junction using nested RT-PCR, followed by Sanger sequencing. Genetic and phylogenetic analyses were performed by comparing VP1-2A sequences with reference sequences and published Tunisian circulating strains.

Viral RNA was detected in 18 (58.1%) of the 31 serum samples that tested positive for HAV IgM using real-time RT-PCR. The VP1-2A junction was successfully sequenced in 13 of these samples. Phylogenetic analysis confirmed the circulation of genotype I, with two subtypes identified. Subtype IA was the most prevalent, found in 12 patients (92.3%), while subtype IB was detected in one patient (7.7%). A phylogenetic study revealed two major variants within subtype IA when compared with published Tunisian sequences. Variant 1 exhibited at least five subvariants, and variant 2 could be divided into three subvariants. Eight of the 12 analyzed sequences belonged to variant 1, and six of these clustered within the same subvariant. The remaining four sequences were classified as variant 2, specifically subvariant 2C. These findings suggest a decline in genetic diversity, possibly due to immune pressure resulting from the introduction of the hepatitis A vaccine (HAV).

A more extensive genetic analysis based on a larger population is essential to gain a deeper understanding of HAV genetic variability in Tunisia and support decision-making regarding the administration of the HAV vaccine.

# Research article: Recherche des entérovirus poliomyélitiques et non poliomyélitiques chez les cas de paralysie flasque aiguë et leurs contacts en Tunisie

**Published:** 2025-11-09

**Authors:** W. Laouej, S. Haddad-Boubaker, I. Ben Salem, H. Hannachi, H. Touzi, Z. Meddeb, W. Hammemi, A. Sadraoui, N. Hogga, M. Yahyaoui, I. Ben Mrad, H. Triki

**Affiliations:** 1 Laboratoire de virologie clinique, Institut Pasteur de Tunis. Laboratoire de référence régional OMS pour la poliomyélite et la rougeole, 1002 Tunis, Belvédère, Tunisie; 2 Laboratoire de recherche Virus, Vecteurs, Hôtes LR20IPT02, Institut Pasteur de Tunis, Tunisie; 3 Université Tunis El Manar, Faculté de médecine de Tunis, 1006, Tunisie; 4 Direction des soins de santé de base, Ministère de la santé, 1006 Tunis, Tunisie

La surveillance virologique des entérovirus (EV) est un pilier essentiel du programme mondial d’éradication du poliovirus, lancé par l’Organisation mondiale de la santé en 1988. Cette surveillance porte principalement sur les cas des paralysies flasques aiguës (PFA) et leurs contacts, visant la détection des entérovirus poliomyélitiques et non poliomyélitiques.

Ce travail a porté sur l’étude du profil des entérovirus poliomyélitiques (PV) et non poliomyélitiques (EVNP) chez des patients atteints de PFA et leurs contacts enregistrés entre janvier 2023 et décembre 2024.

Il s’agit d’une étude rétrospective menée sur 482 échantillons de selles collectés auprès de cas de PFA et de leurs contacts sains entre janvier 2023 et décembre 2024. La détection a été réalisée par isolement viral à partir des cultures cellulaires sur des lignées de rhabdomyosarcome humain et des lignées cellulaires transgéniques de souris exprimant le gène du récepteur cellulaire humain pour le PV (L20B).

Le typage moléculaire des EVNP a été réalisé par séquençage partiel ou complet du gène VP1 utilisant la technologie Sanger. Les sérotypes d’EV ont été assignés en fonction du pourcentage d’identité de leurs séquences avec les séquences du sérotype le plus proche, réalisé par le logiciel BLAST.

Au total, 311 patients ont été inclus dont 164 cas de PFA et 147 contacts sains. Un EV a été isolé chez dix patients (3,2%, 10/311), dont huit cas de PFA (4,8%, 8/164) et deux contacts sains dont un associé à un cas de PFA négatif. Les taux de positivité obtenus sont en concordance avec l’épidémiologie locale décrite entre 2007-2017. L’analyse des séquences a montré que les souches détectées appartenaient aux groupes EV-B (E6, E9, E11, E13, E14 et E30, Coxsackievirus CVB5) et EV-C (PV souche Sabin-like de type 3). Il s’agit des sérotypes qui ont circulé depuis 1992. Tous les cas de PFA associés aux EVNP ont présenté un déficit moteur des membres inférieurs avec abolition des reflex ostéotendineux sans atteinte sensitive. Un tableau de méningite concomitante a été décrit chez un cas de PFA à Echovirus E30. La souche vaccinale Sabin-like du poliovirus de type 3 a été isolée chez un enfant de 6 ans qui a présenté, quatre jours après l’administration de la dose de rappel du vaccin antipoliomyélitique oral, des troubles de comportements dans un contexte fébrile sans signes de myélite associés. La détection des EV en Tunisie est de faible prévalence avec une diversité de sérotypes conforme au profil épidémiologique local. Cependant, le risque des manifestations neurologiques sévères justifie le renforcement du système de surveillance de ces agents.

# Research article: Genetic diversity and excretion kinetics of enteroviruses excreted by patients with primary immunodeficiency in Tunisia

**Published:** 2025-11-09

**Authors:** I. Ben Salem, H. Khemiri, N. Driss, N. Makki, I. BenFraj, I. Ben Mrad, M. Yahyaoui, H. Touzi, Z. Meddeb, I. Ben Mustapha, A. Gzara, M. Ouederni, M. Barbouche, H. Triki, S. Haddad-Boubaker

**Affiliations:** 1 Laboratory of Clinical Virology, WHO Regional Reference Laboratory for Poliomyelitis and Measles for the EMR, Institute Pasteur of Tunis, University of Tunis El Manar, Tunis, Tunisia; 2 Laboratory of Virus, Host and Vectors (LR 20 IPT 02), Institute Pasteur of Tunis, University of Tunis El Manar, Tunis, Tunisia; 3 National Program of Immunization Basic Health Care Division, Ministry of Health, Tunis, Tunisia; 4 Laboratory of Transmission, Control and Immunology of Infections (LR11IPT02), Department of Immunobiology of infections, Institut Pasteur de Tunis, University of Tunis El Manar, Tunis, Tunisia; 5 National Bone Marrow Transplantation Center, Pediatric Hematology-Immunology Unit, Tunis, Tunisia; 6 Primary Health Care Directorate, Ministry of Health, Tunisia; 7 Faculty of Medicine, University of Tunis El Manar, Tunis, Tunisia; 8 Department of Microbiology, Immunology and Infectious Diseases, College of Medicine and Medical Sciences, Arabian Gulf University, Manama, Bahrain

Enteroviruses (EVs) are small, non-enveloped RNA viruses that cause a wide range of clinical manifestations, which are more severe in individuals with primary immunodeficiency (PID). This study examined the genetic diversity and excretion kinetics of EVs in 138 Tunisian patients with PID over a four-year period (2020-2024).

A total of 558 stool samples were collected at monthly intervals for positive results and after six months for negative ones. EV detection was performed by isolating the virus on RD and L20B cell lines, followed by intratypic differentiation (ITD) using multiplex RT-qPCR. Molecular typing was performed using Sanger sequencing of the VP1 region, followed by BLAST analysis. A total of 27 excretion events occurred in 24 EV excreters. Three patients experienced three distinct shedding events, and the remaining 21 patients exhibited continuous viral excretion until clearance. Excretion periods varied from 30 to 946 days. The majority (26/27) exhibited shedding periods ranging from one to six months, while one patient displayed prolonged excretion. Thirteen different EV serotypes were identified in total. One patient was co-infected with E25 and E13, and six patients exhibited continuous excretion of different serotypes at various time points.

This study highlights the diversity of EVs excreted by PID patients and provides insight into EVs circulating in Tunisia. It also underscores the importance of continuous surveillance to prevent potential public health risks, particularly in regions where poliovirus eradication efforts are ongoing.

# Research article: Épidémiologie des infections respiratoires en réanimation pédiatrique à Tunis

**Published:** 2025-11-09

**Authors:** W. Laouej, S. Khedher, K. Meftah, A. Miraoui, A. Bouafsoun, K. Menif, H. Smaoui

**Affiliations:** 1 Service de laboratoire de microbiologie, Hôpital d’enfants Béchir Hamza de Tunis, Tunisie; 2 Université Tunis El Manar, Faculté de médecine de Tunis, LR18ES39, Tunisie; 3 Réanimation médicale pédiatrique, Hôpital d>enfants Béchir Hamza de Tunis, Tunisie

Les infections respiratoires virales sont fréquentes chez l’enfant et concernent tout particulièrement les nourrissons. Ces infections représentent toujours un défi pour nos systèmes de santé pour absorber ce surcroît d’activité saisonnière.

L’objectif de cette étude était de décrire le profil des virus incriminés dans les infections respiratoires de l’enfant en milieu de réanimation à l’hôpital d’enfants Béchir Hamza de Tunis (HEBHT).

Il s’agit d’une étude descriptive rétrospective, ayant inclus les enfants hospitalisés pour un syndrome respiratoire aigu sévère au service de la réanimation pédiatrique à l’HEBHT entre mars 2019 et mars 2024. Une PCR respiratoire multiplex (Respifinder^®^, Pathofinder; Biofire^®^, bioMérieux; QIAstat^®^, Qiagen) et/ou une RT-PCR Covid-19 et/ou une recherche de l’antigène viral du virus respiratoire syncytial (VRS) par un test immunochromatographique ont été réalisées sur les aspirations nasopharyngées et les lavages broncho-alvéolaires.

Au total, 1 141 enfants ont été inclus. Une prédominance masculine a été notée avec un sexratio à 1,57 (387/245). La majorité des enfants inclus étaient des nourrissons de moins de 1 an (n=565; 88,98%) et 24,88% (n=158) étaient des nouveaux nés. Au moins un virus a été détecté dans 64,3% des cas (n=635) dont 92,44% ont été détectés par PCR multiplex (n=587). La répartition saisonnière a montré des pics en hiver (n=292; 46%) et en automne (n=168; 26%). Le VRS était le principal virus isolé (n=341; 54%) suivi par le Rhinovirus-Entérovirus (n=165; 26%) et le SARS-CoV2 (n=63; 10%). Durant l’année 2021, le taux de détection virale était maximal. La prévalence de VRS, SARS-CoV2 et RV/EV était respectivement de 57%, 34% et 23%. Les pourcentages de détection de Bocavirus, Human Metapneumovirus et Parainfluenza Virus étaient également les plus élevés en 2021 avec respectivement (n=18/30,60%), (n=18/36,50%), (n=19/43,44%).

Malgré la flambée de la Covid-19 durant cette période, la prévalence d’autres virus respiratoires a pris le dessus. Les mesures strictes prises pour limiter ce fléau étaient insuffisantes, d’où l’importance de la surveillance épidémiologique et l’élargissement du spectre d’action.

# Research article: Association entre le polymorphisme de l’interleukine-10 et l’infection chronique par le virus de l’hépatite B

**Published:** 2025-11-09

**Authors:** Y. Chelbi, M. Hamdoun, H. Cherni, I. Fhoula, O. Bahri

**Affiliations:** Laboratoire de microbiologie-biochimie, Hôpital Aziza Othmana, Tunis, Tunisie

L’infection chronique par le virus de l’hépatite B (VHB) demeure un problème majeur de santé publique. Elle est fréquente et peut se compliquer de cirrhose et de carcinome hépatocellulaire (CHC). Son évolution dépend de plusieurs facteurs notamment immunogénétiques.

L’objectif de cette étude était d’étudier l’association entre le polymorphisme de l’IL-10 et l’évolution de ce type d’infection.

Il s’agissait d’une étude rétrospective de type cas-témoin portant sur 156 porteurs chroniques du VHB (PC-VHB) et 174 témoins sains non infectés par le VHB (AgHBs-). L’étude du polymorphisme allélique de l’IL-10 a été réalisée par la technique de discrimination allélique type TaqMan au niveau de deux « *single nucleotide polymophism* » (SNPs) -592A/C et -1082A/G du promoteur du gène IL-10. Le dosage de l’IL-10 a été fait par la technique de type ELISA « maison » pour tous les patients infectés chroniques du VHB n’ayant pas encore bénéficié de traitement. L’âge moyen des patients était de 46 ans. Une infection chronique AgHBe (-) (ICB) était retenue dans 32% (n=43) des cas; 37% (n=51) des patients avaient une hépatite chronique active et 31% une hépatite compliquée (34 cirrhotiques et 7 CHC). L’étude du polymorphisme allélique a identifié 6 génotypes différents: AA (14%), AC (43%) et CC (43%) pour le SNP-592A/C et AA (41%), AG (45%) et GG (14%) pour le SNP-1082A/G. Seul le génotype -1082/GG était significativement plus fréquent chez les PC-VHB (OR=1,9; IC95% = [1-3,62]; p=0,046). Le taux d’IL-10 était significativement plus élevé en cas d’ICB comparé aux témoins (3,27 vs 2,56 pg/ml; p=0,002). Les génotypes -1082 GG (6,02 pg/ ml; p=0,04) et -592CC (3,73 pg/ml; p=0,039) étaient associés à des taux significativement plus élevés d’IL-10. Ce taux était significativement plus faible avec le génotype -592 AA (1,35 pg/ ml; p=0,014).

Ces résultats confirment que les taux élevés d’IL-10 et le polymorphisme -1082 GG du promoteur de son gène sont associés à l’évolution vers la chronicité de l’infection par le VHB.

# Research article: Profil microbiologique des infections respiratoires virales diagnostiquées par approche syndromique moléculaire

**Published:** 2025-11-09

**Authors:** N. Assali, S. Mhalla, R. Ben Cheikh, A. Nabli, M. Mastouri

**Affiliations:** Laboratoire de microbiologie, Hôpital Fattouma Bourguiba, Monastir, Tunisie

Les infections respiratoires virales représentent un enjeu majeur de santé publique particulièrement depuis la pandémie de SARS-CoV-2. Le diagnostic étiologique permet de préciser leur épidémiologie microbiologique locale afin de mettre au point une stratégie préventive adaptée. L’objectif de ce travail est de dresser le profil microbiologique des infections respiratoires et les caractéristiques des patients diagnostiquées par approche syndromique moléculaire.

Nous avons analysé tous les prélèvements respiratoires parvenus au laboratoire dans le cadre de l’exploration d’une infection respiratoire par approche syndromique moléculaire entre janvier 2021 et février 2025. La détection des germes a été réalisée par une technique de PCR multiplex en temps réel (RespiFinder^®^ 2SMART) qui permet de détecter jusqu’à 24 virus et bactéries respiratoires dans un même test.

Nous avons colligé 456 prélèvements non redondants. Un total de 163 tests (35,6%) avait au moins un virus détectable par PCR multiplex. L’âge moyen des patients était de 4 ans [5 mois - 100 ans] et le sex-ratio (H/F) de 1,7. La majorité des demandes provenait du service de pédiatrie (57,1%) suivi du service de réanimation médicale (24,5%). Chez les enfants (<14 ans), l’analyse du profil microbiologique a montré une prédominance de VRS, isolés dans 41% des cas, suivis de Rhinovirus/Entérovirus (15%).

Plusieurs virus ont été retrouvés dans un même prélèvement chez 12% des cas et le virus le plus incriminé était le virus respiratoire syncytial B. Chez les adultes, le profil se caractérise par la prédominance du virus grippaux (notamment celui de la grippe A H1N1) et du coronavirus type 229E retrouvés respectivement dans 19% et 14,3% des cas. Le virus de la grippe dominait également les détections multiples, suivi de rhinovirus/entérovirus (11,1%).

D’après nos résultats, la PCR multiplexe a démontré une circulation virale similaire à celle d’avant l’ère Covid. Il est primordial de ce fait, de promouvoir la vaccination antigrippale et d’aspirer à la disponibilité de celle contre le VRS.

# Research article: Is the Post COVID-19 Condition associated with defective proresolving power?

**Published:** 2025-11-09

**Authors:** A. Bouabdallah, MK. Ben Fradj, MB. Hammami, R. Abdelmalek, L. Ammari, M. Feki

**Affiliations:** 1 Laboratory of Biochemistry, LR99ES11, Rabta Hospital of Tunis, Faculty of Medicine of Tunis, Tunisia; 2 Infectious Diseases Department, Rabta Hospital of Tunis, Faculty of Medicine of Tunis, Tunisia

A post-COVID condition (PCC) is characterized by new or persistent symptoms that appear at least three months after a person has had a case of COVID-19. The underlying mechanisms are unclear. Oxylipins are bioactive lipids involved in the inflammatory response. Arachidonic acid (ARA) produces prostaglandins (PGs) *via* cyclooxygenase (COX) activity, as well as leuko-trienes (LTs) and lipoxins (LXs) *via* lipoxygenase (LOX) activity. PGs and LTs are pro-inflammatory, while LXs are pro-resolving mediators. This study examined the association between oxylipins and SARS-CoV-2 infection during the first week of hospitalization and PCC.

Patients admitted to the Infectious Diseases Department at Rabta Hospital for treatment of a confirmed SARS-CoV-2 infection between January and June of 2021 were included in the study. Plasma oxylipins were analyzed using a targeted LC-MS/MS method on the first and seventh days of hospitalization. At the three-month follow-up after discharge, patients exhibiting symptoms were labelled PCC+, and those without symptoms were labelled PCC-.

Of the 60 patients reviewed at the three-month follow-up, 34 were PCC-and 26 were PCC+. There were no significant differences between the two groups in terms of age, gender, comorbidities, duration of hospital stay, or need for and duration of oxygen therapy. The oxylipin profiles on the first day did not differ significantly between the groups. However, on the seventh day, the PCC+ group had higher levels of LOX-derived LXs and LTs, particularly LXA4 (p = 0.040), 6-epi-LXA4 (p = 0.035), and LXB4.

The study revealed decreased arachidonic acid (ARA) and increased lipoxygenase (LOX) products, including LXs and LTs, in the PCC+ group. While both the COX and LOX pathways are overexpressed in patients with SARS-CoV-2 infection, PCC appears to be associated with a shift in the arachidonic acid (ARA) cascade toward the LOX pathway at the expense of the COX pathway. The increased pro-resolving LXs may indicate an unsuccessful attempt.

# Research article: Epidemiology of respiratory viruses circulating in Tunisia (2022-2025)

**Published:** 2025-11-09

**Authors:** M. Selmi, L. Kanzari, S. Abid, H. El Ghord, L. Charaa, I. Landolsi, A. Ferjani, C. Zedini, I. Boutiba Ben-Boubaker

**Affiliations:** 1 Charles Nicolle Hospital, Laboratory of Microbiology, National Influenza Centre, Tunis, Tunisia; 2 University of Tunis El Manar, Faculty of Medicine of Tunis, LR99ES09, Research Laboratory « Antimicrobial Resistance », Tunis, Tunisia; 3 Primary Healthcare Directorate, Ministry of Health, Tunis, Tunisia

Respiratory infections are one of the biggest public health concerns. Since 2014, a sentinel surveillance program has been in place at the National Influenza Center (NIC) in Tunisia for monitoring influenza viruses.

The aim of this study is to identify circulating respiratory viruses in Tunisia and describe their epidemiological patterns.

From October 2022 to February 2025, nasopharyngeal samples (NPS) were collected from outpatients with influenza-like illness (ILI) and inpatients with severe acute respiratory infection (SARI). The NPS were tested at the NIC at Charles Nicolle Hospital using the CDC multiplex assay and the FTD respiratory pathogens 21 assay.

A total of 6,069 patients were included in the study, 3,199 of whom (52.71%) tested positive for at least one virus. The most frequently detected viruses were enterovirus/rhinovirus (ER) (16.24%), influenza A virus (12.24%), and respiratory syncytial virus (RSV) (11.69%). Influenza A virus and RSV were the most frequent interviral co-infections (n = 423). During the 2022-2023 season, ER was the most prevalent virus (n = 347), followed by influenza A (n = 300) and RSV (n = 261). During the 2023-2024 season, ER cases increased to 412, maintaining its dominance, while influenza A cases rose to 344, and RSV cases reached 294. For the 2024-2025 season, for which data is available from October 2024 to February 2025, ER remained the most prevalent virus (n = 227), followed by RSV (n = 155) and influenza A (n = 99). Seasonal variation was observed. In the fall, ER (45.65%), parainfluenza viruses (1, 2, 3, and 4) (13.33%), and coronaviruses (229E, OC43, NL63, and HKU1) (13.04%) were the most prevalent. The influenza A virus peaked in winter at 22.81%. Spring saw dominance of ER (27.89%), SARS-CoV-2 (14.36%), and metapneumovirus (14.19%). Summer saw ER dominance (32.31%). In conclusion, enterovirus/rhinovirus, influenza A, and respiratory syncytial virus (RSV) were the most common viruses in the last three seasons. Understanding the epidemiology and seasonality of these infections is crucial for planning vaccination and treatment strategies.

# Research article: Apport de l’intelligence artificielle dans l’évaluation des lésions pulmonaires du syndrome de détresse respiratoire aiguë lié au Covid 19: comparaison avec l’expertise radiologique

**Published:** 2025-11-09

**Authors:** W. Nouira, Z. Hammouda, J. Saad, M. Abdelali, M. Lahmar, A. Zaaibi, C. Chelbia, I. Maatouk, E. Bedhiafi, F. Dachraoui, A. Zrig, L. Ouanes Besbes

**Affiliations:** 1 Service de réanimation polyvalente de Monastir, Tunisie; 2 Service de radiologie de Monastir, Tunisie

L’imagerie par tomodensitométrie (TDM) thoracique a joué un rôle central dans le diagnostic de la pneumonie interstitielle chez les patients atteints du syndrome de détresse respiratoire aiguë (SDRA) lié au SARS-CoV-2. Elle a été utilisée pour estimer l’étendue de l’atteinte pulmonaire, soit qualitativement par inspection visuelle, soit quantitativement à l’aide de logiciels semi-automatiques basés sur l’intelligence artificielle (IA). L’objectif de cette étude est d’évaluer la concordance entre l’analyse visuelle qualitative réalisée par des radiologues et le score quantitatif généré par le logiciel semi-automatique Slicer 3D pour estimer l’étendue de l’atteinte pulmonaire dans le SDRA lié au SARS-CoV-2.

Il s’agit d’une étude rétrospective basée sur les données TDM thoraciques de 251 patients présentant un SDRA Covid-19, hospitalisés dans le service de réanimation polyvalente de

l’Hôpital de Monastir entre septembre 2020 et décembre 2022. Tous les patients ont bénéficié d’un scanner thoracique sans injection. L’évaluation qualitative visuelle réalisée par les radiologues a été comparée aux analyses quantitatives obtenues *via* Slicer 3D, en évaluant la concordance entre les deux méthodes (sous-estimation, concordance, surestimation).

L’âge médian des patients était de 59 ans (IQR = [49-67 ans]) et 65% étaient de sexe masculin. La durée médiane de séjour en réanimation était de 10 jours (IQR = [6-15 jours]), avec un taux de mortalité de 41% (n=103). Selon l’analyse des radiologues, 87 patients (34,7%) présentaient des lésions sévères et 82 (32,7%) des lésions critiques, selon la classification de la Société d’imagerie thoracique. L’analyse *via* le logiciel Slicer 3D a identifié des atteintes sévères dans 50% des cas. Une concordance entre les estimations du logiciel et les observations des radiologues a été observée dans 59% des cas, avec une surestimation dans 29% des cas et une sous-estimation dans 12% des cas. La mortalité était significativement corrélée à l’étendue des lésions pulmonaires observées par les radiologues à la TDM (p<0,001). Par ailleurs, les calculs du logiciel Slicer 3D montraient une mortalité associée à un volume pulmonaire total significativement plus réduit (2 217 ml contre 2834 ml; p<0,001) et à un pourcentage d’atteinte parenchymateuse plus élevé (72% contre 61%; p<0,001).

Notre étude met en évidence une bonne corrélation entre l’évaluation qualitative des radiologues et les analyses quantitatives fournies par le logiciel Slicer 3D. Par ailleurs, l’étendue des lésions parenchymateuses et la volumétrie pulmonaire sont significativement associées au pronostic des patients atteints de SDRA lié au Covid-19. Ces résultats suggèrent que l’intelligence artificielle, en tant qu’outil collaboratif, constitue un soutien précieux dans l’analyse et l’interprétation des images médicales, en particulier dans des contextes critiques comme le SDRA.

# Research article: Dépistage et répartition génotypique des papillomavirus humains chez une population de femmes tunisiennes et impact sur la vaccination anti-HPV

**Published:** 2025-11-09

**Authors:** A. Youssfi, ML. Ben Moussa, G. Njoumi, I. Chabbar, M. Ben Moussa

**Affiliations:** Laboratoire de virologie, Hôpital militaire principal d’instruction de Tunis, Tunisie

Les papillomavirus humains (HPV) sont responsables de l’infection sexuellement transmissible la plus fréquente. Certains de ces HPV sont oncogènes, induisant surtout le cancer du col de l’utérus. Actuellement, une vaccination adaptée permet de prévenir l’infection par certains HPV oncogènes. Cette étude a pour but d’analyser la prévalence et la répartition génotypique d’HPV chez une population féminine tunisienne recrutée en 2024 et issue de différentes régions du pays. Cette étude multicentrique rétrospective a été menée au sein du laboratoire de virologie de l’Hôpital militaire principal d’instruction de Tunis entre mai 2024 et février 2025. Des prélèvements cervico-vaginaux étaient réalisés à l’aide d’une cytobrosse au niveau de l’endocol et déchargés dans le milieu de transport et de conservation CareHPV Collection Medium®. La détection du HPV était faite par une RT-PCR utilisant le kit Neu MoDx™ HPV Test Strip (Qiagen). Tous les prélèvements positifs étaient génotypés par RT-PCR à l’aide du kit Anyplex II HPV28 Detection (Seegene) permettant de détecter 28 génotypes de HPV dont 19 oncogènes. Cette étude a inclus 1 719 femmes présentant des âges extrêmes allant de 21 à 75 ans. La PCR était positive chez 56 femmes, soit une prévalence de 3,25%. Le génotypage effectué pour les prélèvements positifs a montré une répartition génotypique très hétérogène. En effet 51,8% des génotypes étaient oncogènes (16,18,31,33,35,45,51,52,53,58,59,68) dont les plus fréquents étaient les génotypes 52,33 et 16 avec des pourcentages respectifs de 10,7%, 8,9% et 7,1%. D’autres génotypes à haut risque (53,31,68 et 45) étaient également isolés avec des pourcentages allant de 3,6% à 1,8%. Le complexe 52 33 35 58 était détecté dans 7,1% des cas d’HPV positifs. Bien que la prévalence de l’infection par le HPV soit faible en Tunisie, la stratégie vaccinale devrait couvrir les HPV oncogènes les plus présents dans notre population comme les types 52 et 33 et ceci grâce au vaccin nanovalent recommandé actuellement.

# Research article: Contribution de la PCR sur extraits de frottis dermiques au diagnostic de la leishmaniose cutanée

**Published:** 2025-11-09

**Authors:** F. Maatallah, W. Aïssi, N. Boulehmi, O. Souissi, M. Zerzeri, Ch. Fouzai, R. Ben Abdallah, E. Siala, A. Bouratbine, K. Aoun

**Affiliations:** Laboratoire de parasitologie-mycologie, LR 20-IPT-06, Institut Pasteur de Tunis, Tunisie

Le diagnostic de la leishmaniose cutanée (LC) repose encore sur la mise en évidence du parasite à l’examen direct (ED). Bien que cette technique soit simple et peu coûteuse, la PCR est actuellement décrite comme plus sensible.

L’objectif de notre étude était d’estimer la contribution d’une technique de PCR en temps réel dans le diagnostic de la LC.

Deux-cent vingt-sept frottis dermiques archivés correspondant à des patients suspects de LC adressés au laboratoire de Parasitologie de l’Institut Pasteur de Tunis ont été examinés au microscope optique pendant 20 minutes puis grattés, et l’ADN extrait à l’aide du kit QIAamp DNA Blood Mini Kit, Qiagen. Les extraits ont été testés en PCR en temps réel par la technologie TaqMan selon un protocole mis au point dans notre laboratoire. La charge parasitaire (CP) de chaque échantillon a été calculée par rapport à une gamme standard d’extraits de promastigotes préparés en culture et quantifiés.

Le diagnostic de LC a été retenu pour 101 patients. L’ED était positif dans 76 cas et la PCR dans 100 cas. La sensibilité de la PCR (99%) était significativement supérieure à celle de l’ED (75,3%) (p<0,001). La PCR a permis de rattraper 25 patients (24,8%) faussement négatifs à l’ED. La charge parasitaire en PCR était très différente d’un frottis à l’autre. Elle a varié entre 0,1 et 34 898,5 parasites avec une médiane de 2,5 parasites par échantillon. En comparant les valeurs des CPs dans les 2 groupes ED positif et ED négatif, la moyenne était significativement supérieure dans le 1^er^ groupe (1 213,1 parasites *versus* 15,2) (p=0,012); 44% des frottis négatifs comportant 1 parasite ou moins.

La meilleure sensibilité de la PCR, notamment sur les frottis à faibles CP, se confirme dans notre étude. La PCR sur frottis colorés représente ainsi une alternative prometteuse pour le diagnostic de la LC. Sa pratique sur les frottis colorés est performante et permet d’éviter de reprélever les patients.

# Research article: Le kyste hydatique du foie: étude prospective des facteurs prédictifs de la fertilité et de la viabilité du liquide hydatique. A propos de 126 cas

**Published:** 2025-11-09

**Authors:** R. Zouari, I. Kharrat, R. Daoued, S. Saumtally, F. Cheikhrouhou, A. Ayadi, A. Trigui, S. Boujelbene

**Affiliations:** Service de chirurgie générale, CHU Habib Bourguiba, Sfax, Tunisie. Laboratoire de parasitologie, CHU Habib Bourguiba, Sfax, Tunisie

L’échinococcose kystique hydatique, endémique en Tunisie, est un problème de santé publique. Sa prise en charge repose sur la classification de l’OMS, basée sur l’activité des kystes. Actuellement, aucun critère clinique ne permet de prédire la viabilité du liquide hydatique (LH). L’objectif était de déterminer la fertilité et la viabilité des protoscolex du LH des kystes hépatiques humains et d’identifier les facteurs prédictifs de ces deux paramètres.

Il s’agit d’une étude prospective analytique portant sur les kystes hydatiques du foie (KHF) opérés au service de chirurgie générale du CHU Habib Bourguiba de Sfax entre mars 2018 et décembre 2023.

L’analyse de la fertilité et de la viabilité des protoscolex du LH a été réalisée au laboratoire de parasitologie-mycologie. La fertilité était définie par la présence de protoscolex ou de crochets dans le LH, tandis que la viabilité correspondait au nombre de protoscolex viables au sein du kyste.

L’étude a analysé 126 KHF de 70 patients, avec un taux de fertilité de 71,4% (90 kystes). En analyse bivariée, les facteurs prédictifs de fertilité étaient: âge > 35 ans, genre féminin, aspartate aminotransférase < 21 UI/L, aspect en eau de roche et compression vasculaire. Les facteurs associés à l’absence de fertilité étaient les kystes CE4 et le liquide gélatineux. En analyse multivariée, les facteurs prédictifs de fertilité étaient: âge avancé (OR=1,047), compression vasculaire (OR=3), types CE1 (OR=11,6) et CE2 (OR=15,94). La médiane du nombre de protoscolex viables était de 1,5. Les facteurs de viabilité étaient: type CE1, liquide en eau de roche et compression vasculaire. Les facteurs de non-viabilité incluaient CE3b, CE4, calcifications pariétales et liquide gélatineux.

L’évaluation de la fertilité du LH et de la viabilité des protoscolex permet de distinguer les kystes actifs des kystes inactifs, influençant ainsi la prise en charge thérapeutique, qu’elle soit chirurgicale, percutanée ou médicamenteuse.

# Research article: Facteurs prédictifs de fertilité des vésicules filles dans l’échinococcose kystique du foie: étude prospective à propos de 248 cas

**Published:** 2025-11-09

**Authors:** R. Zouari, I. Kharrat, R. Daoued, W. Hentati, W. Boujelbene, S. Saumtally, F. Cheikhrouhou, A. Ayadi, A. Trigui, S. Boujelbene

**Affiliations:** Service de chirurgie générale, CHU Habib Bourguiba Sfax, Tunisie. Laboratoire de parasitologie CHU Habib Bourguiba, Sfax, Tunisie

Le cycle *d’Echinococcus granulosus* et son potentiel évolutif a été toujours un sujet de débat. Ainsi, si l’origine des protoscolex était bien définie, l’origine des vésicules filles (VF) n’était pas claire et plusieurs hypothèses ont été proposées. L’objectif de ce travail était d’étudier la fertilité des VF et ses facteurs prédictifs afin d’en déterminer l’origine.

Il s’agissait d’une étude pilote prospective incluant les échinococcoses kystiques du foie (EKF) contenant des vésicules filles opérés au service de chirurgie générale et viscérale du CHU Habib Bourguiba de Sfax pendant une période de 22 mois. Une étude parasitologique des VF incluant la détermination de leur fertilité a été réalisée au laboratoire de parasitologie.

Un total de 248 VF prélevés de 27 kystes multivésiculaires provenant de 21 patients a été inclus dans notre étude. Le taux de fertilité des VF était de 64,5%. Des crochets étaient présents dans 130 VF (52,4%). En analyse multivariée, seules les transaminases > 18 UI/L, les bilirubines totales > 8,5 gmol/L, le type CE2 de la classification OMS, la taille des kystes > 47 mm, le nombre de VF/ kyste > 4 et la taille des VF > 6,5 mm étaient des facteurs significativement associés à la fertilité des VF (p<0,05). L’âge et les antécédents d’EKF opérées ont été des facteurs significativement associés à la fertilité des VF (p<0,05).

Les vésicules filles peuvent être fertiles. Elles pourraient avoir pour origine la membrane proligère ou le protoscolex invaginé. Le protoscolex évaginé est une forme abortive et pourrait être un phénomène dégénératif après tentative de vésiculation caudale.

# Research article: Intelligence artificielle et diagnostic du paludisme, une solution adaptée aux hôpitaux tunisiens

**Published:** 2025-11-09

**Authors:** S. Aissa, D. Dhouib, D. Aloui, M. Bouchekoua, S. Trabelsi, F. Ghorbell

**Affiliations:** 1 Groupe de recherche Images et Formes de Tunisie (GRIFT), Laboratoire CRISTAL, Manouba, Tunisie; 2 Laboratoire de parasitologie-mycologie, Hôpital Charles Nicolle, Tunis, Tunisie

Le diagnostic du paludisme repose sur l’examen microscopique des frottis sanguins et des gouttes épaisses, une méthode chronophage et sujette à l’erreur humaine. La qualité sous-optimale des lames, liée à une confection ou une coloration mal faites, aggrave ce problème.

Cette étude propose une solution basée sur l’intelligence artificielle (IA), développée par le laboratoire CRISTAL en collaboration avec le centre de parasitologie de l’Hôpital Charles Nicolle, afin d’améliorer la précision et l’efficacité du diagnostic.

Nous avons développé un système d’IA en deux étapes. La première repose sur une segmentation des globules rouges afin d’éliminer les régions non pertinentes. La seconde exploite des techniques d’analyse d’image pour extraire des caractéristiques distinctives de chaque cellule, suivies d’un mécanisme de classification par vote. Ce système a été conçu à partir d’images issues de l’Hôpital Charles Nicolle, permettant ainsi d’adapter l’algorithme aux spécificités des frottis tunisiens. Sa performance a été évaluée par validation croisée sur des échantillons locaux et externes afin d’en garantir la robustesse.

Les tests effectués sur des données du site des *National Institutes of Health,* comprenant 27 000 cellules (infectées et saines), ont montré une précision globale d’environ 74,6%. Notamment, le système traite chaque échantillon en moins de 5 secondes, contre 15 à 30 minutes pour l’examen manuel. Bien que le modèle identifie efficacement les cellules non parasitées avec 490 faux positifs, le nombre plus élevé de faux négatifs (688) pour les cellules infectées indique une tendance à sousclasser ces dernières.

Cette approche basée sur l’IA constitue une avancée prometteuse pour la détection du paludisme en milieu clinique tunisien. En automatisant l’analyse des frottis sanguins, elle réduit les délais diagnostiques et allège la charge des laboratoires. Malgré certaines limites, nos résultats suggèrent que des solutions d’IA ciblées peuvent répondre aux défis de la santé locale sans modifier les pratiques de prélèvement. Ces résultats justifient une évaluation clinique approfondie.

# Research article: Enquête de l’évaluation du contrôle national de la qualité en parasitologie en Tunisie

**Published:** 2025-11-09

**Authors:** Ch. Ibrahim, H. Bouguerra, Ch. Hamrita, K. Sakka, R. Meddeb-Asli, M. Mejri, H. Battikh

**Affiliations:** 1 Unité des laboratoires de biologie médicale (ULB), ministère de la Santé, Tunis, Tunisie; 2 Direction des Études et de la planification, ministère de la Santé, Tunis, Tunisie; 3 Faculté de médecine de Tunis, Université Tunis-El-Manar, Tunisie

Le contrôle national de qualité (CNQ) dans les laboratoires d’analyses médicales, préconisé par la législation tunisienne et supervisé par le ministère de la Santé, constitue un outil précieux qui contribue à garantir la fiabilité, la précision et la sécurité des analyses médicales en vue de fournir des résultats de haute qualité aux professionnels de santé et aux patients. Dans cette étude, nous avons réalisé une enquête rétrospective sur le CNQ en parasitologie en Tunisie, à l’attention de 750 laboratoires concernés par le CQN des laboratoires d’analyses médicales publics et privés, sur la période 2016-2023. L’évaluation a porté sur trois tests parasitologiques: l’examen parasitologique des selles (coprologie parasitaire), la recherche de parasites dans les frottis sanguins et la recherche de parasites dans les frottis cutanés. Les principaux résultats de cette enquête montrent que le taux de conformité pour le test de coprologie parasitaire était supérieur à 75% durant les quatre premières années de l’étude (2016-2019). Cependant, une baisse progressive a été observée, atteignant 52% au cours des deux dernières années (2019-2023). En ce qui concerne la recherche des parasites sur frottis sanguins et cutanés, les taux de conformité ont varié entre 59% et 91% au cours des cinq premières années de l’étude (2016-2021), témoignant de performances globalement satisfaisantes. En conclusion, bien que les résultats de conformité restent globalement satisfaisants pour l’ensemble des épreuves parasitologiques, la baisse observée pour certains tests souligne la nécessité d’un renforcement des actions de formation et d’amélioration continue des pratiques analytiques au sein des laboratoires. Ces résultats reflètent à la fois la rigueur des méthodes employées et l’importance du CNQ dans l’assurance qualité des analyses médicales en Tunisie.

# Research article: Early ventilator-associated pneumonia in trauma patients: epidemiology and prognostic impact

**Published:** 2025-11-09

**Authors:** I. Bannour, R. Jaafer, N. Mabrouk, O.K. Gardeb-bou, A. Maatoug, H. Attia, M. Ouaz, K. Mas-moudi, A. Majdoub

**Affiliations:** Service d’anesthésie réanimation, EPS Taher Sfar, Mahdia, Tunisie

Patients with polytrauma have impaired innate and acquired immunity, increasing their risk of nosocomial infections.

Our study aimed to determine the epidemiological and bacteriological profile of early ventilator-associated pneumonia (VAP) in trauma patients, as well as its impact on prognosis.

We conducted a retrospective study over two years (January 2021-December 2022). We included all trauma patients over the age of 15 who required mechanical ventilation for more than 48 hours. We collected each patient’s epidemiological, clinical, biological, radiological, and therapeutic data. We compared the variables using appropriate statistical tests, including chi-square, Fisher’s exact, and Student’s t-tests. A p-value less than .05 was considered significant. Statistical analyses were performed using SPSS Statistics version 22.

During this period, 105 patients were admitted to our resuscitation unit, 37 of whom were intubated either immediately or within the first 48 hours. The mean age was 30.81 ± 17.68 years. The mean APACHE II, IGS II, and ISS scores were 14.24 ± 6.98, 28.35 ± 14.07, and 32.22 ± 15.2, respectively. Twenty-five patients (67.5%) had chest trauma, and 28 patients (75.7%) had head trauma. Among the patients with head trauma, 17 (45.9%) had a GCS of less than 8 and 17 (45.9%) developed ventilator-associated pneumonia (VAP). *Pseudomonas aeruginosa* caused 9.2% of infections (23.5% of VAP cases), followed by *Acinetobacter baumannii* and *Staphylococcus aureus* (17.6% of VAP cases). *Klebsiella pneumoniae, E. coli, Proteus mirabilis* and Enterobacter aerogenes were each isolated in 5% of VAP cases.

# Research article: Épidémiologie des infections associées aux soins dans le service de neurochirurgie au CTGB

**Published:** 2025-11-09

**Authors:** S. Zelfani, S. Dhraief, O. Faten, I. Zammel, L. Thabet

**Affiliations:** 1 Laboratoire de biologie médicale, Centre de traumatologie et des grands brûlés, Université Tunis el Manar, Faculté de médecine de Tunis, UR22SP03, Tunisie; 2 Service d’anesthésie-réanimation, Centre de traumatologie et des grands brûlés de Ben Arous, Tunisie; 3 Service de neurochirurgie, Centre de traumatologie et des grands brûlés de Ben Arous, Tunisie

Les infections associées aux soins (IAS), communément appelées infections nosocomiales posent un problème majeur de santé publique en raison de leur impact sur la morbidité, la mortalité et les coûts associés aux soins.

L’objectif de cette enquête était d’évaluer la fréquence et la nature des IAS dans le service de neurochirurgie, ainsi que l’évolution clinique des patients.

Il s’agit d’une étude rétrospective menée sur une période de trois mois (1^er^ avril-30 juin 2024) incluant les malades hospitalisés au service de neurochirurgie au Centre de traumatologie et des grands brûlés ayant développés une IAS. Les dossiers des malades étaient consultés avec remplissage d’une fiche de renseignement. L’identification bactérienne était faite par les méthodes conventionnelles et l’étude de la sensibilité aux antibiotiques était effectuée selon les recommandations du Comité de l’antibiogramme de la Société française de microbiologie 2024.

Sur un total de 199 patients hospitalisés, 7,5% des patients présentaient une IAS (n=15). L’âge moyen était de 56 [extrêmes: 28-76] ans. Le sexratio (H/F) était 0,6. Selon le siège de l’infection, les infections pulmonaires étaient les plus fréquentes (34%), suivies des infections urinaires (28%), des bactériémies (16%), des infections méningées (8%) et d’autres infections diverses (14%). Au total, 52 prélèvements bactériologiques positifs étaient colligés avec identification de 16 bactéries multirésistantes (BMR) soit 32% des prélèvements. Parmi les 16 souches de BMR, *Acinetobacter baumannii* et *Klebsiella pneumoniae* étaient isolées à des taux de 37,5% chacune. *Enterobacter cloacae* était identifié dans 12,5% des cas. *Enterococcus faecium* et *Staphylococcus aureus* résistant à la méticilline étaient isolées à des taux similaires de 6,25% chacune. Une souche de *K. pneumoniae* résistante à la colistine ainsi qu’une souche d’E. *faecium* résistante à la vancomycine (ERV) étaient notées. Les traitements anti-infectieux à large spectre employés étaient: linézolide (n=9), colistine (n=8), pénicillines à large spectre (n=5) et carbapénèmes (n=4). La durée moyenne d’hospitalisation était de 35 jours. L’évolution des patients infectés était défavorable dans 40% des cas (n=6). Cinq décès étaient recensés à la suite de l’IAS.

La prévalence élevée (7,5%) des IAS avec une évolution défavorable dans 40% des cas et survenue de cinq décès impose des mesures urgentes pour contrôler ces infections. Le réaménagement du bloc opératoire avec séparation du bloc septique du bloc non septique devient une nécessité.

# Research article: Pneumopathie acquise sous ventilation mécanique chez les brûlés à germes non fermentant: incidence et facteurs associés.

**Published:** 2025-11-09

**Authors:** H. Fredj, L. Borsali, A. Aloui, A. Alouini, Z. Mahjoubi, M. Sghaier, S. Dhraief, I. Jami, B. Gasri, L. Thabet, AA. Messadi, A. Mokline

**Affiliations:** 1 Service de réanimation des brûlés, centre de traumatologie et des grands brûlés, Ben Arous, Tunisie; 2 Laboratoire de biologie médicale et banque du sang, centre de traumatologie et des grands brûlés, Ben Arous, Tunisie

Les pneumonies acquises sous ventilation mécanique (PAVM) à bacilles à Gram négatif non fermentant sont associées à une morbi-mortalité importante.

L’objectif de l’étude était de déterminer l’incidence, les facteurs associés à la survenue et à la mortalité de PAVM à germes non fermentant chez le brûlé par une étude rétrospective menée sur une période de deux ans (2022-2023) dans le service de réanimation des brûlés en Tunisie. Ont été inclus les patients adultes brûlés ayant développé une PAVM à germes non fermentant. La PAVM a été retenue selon les critères de la Société française d’anesthésie et de réanimation. Afin de déterminer les facteurs associés, ce groupe a été comparé à un groupe de patients ayant une PAVM à autre germe.

Pendant la période d’étude, 856 patients ont été hospitalisés, 214 (25%) ont nécessité une ventilation mécanique invasive, 64 ont présenté au moins un épisode de PAVM dont 47 étaient dues à un germe non fermentant (70%). L’âge moyen des patients était de 38±14 ans avec un sex-ratio de 3,7. La majorité était transférée d’un autre hôpital (n=37,79%). Les brûlures étaient thermiques dans 91% des cas, secondaires à une tentative de suicide par immolation dans 57% des cas. La surface cutanée brûlée moyenne était de 41±20%. La PAVM était polymicrobienne dans 72% des cas.

Les germes incriminés étaient: *Acinetobacter baumannii* (62% des cas), *Pseudomonas aeruginosa* (59%), *Pseudomonas fluorescens* (8,5%) et *Stenotrophomonas maltophilia* (2%). Les facteurs associés à la survenue de cette complication étaient la présence de brûlures pulmonaires (p=0,04), un délai de transfert supérieur à 48 h (p=0,03), l’âge (p=0,05), la SCB (p=0,04), les scores ABSI (p=0,005) et IB (p=0,003). La PAVM était compliquée d’état de choc septique dans 95% des cas et de syndrome de détresse respiratoire aigu dans 57% des cas. L’évolution était favorable dans 47% des cas. Une récidive était observée dans 32% des cas. La mortalité était de 53%.

En conclusion, la PAVM à germes non fermentant est fréquente chez le brûlé, les germes les plus incriminés sont *A. baumannii* et *P. aeruginosa*. Elle est associée à une mortalité importante.

# Research article: Profil des résistances du VIH-1 aux antirétroviraux en Tunisie

**Published:** 2025-11-09

**Authors:** N. Kouched, Z. Bouslah, S. Abid, H. Limam, S. Lachtar, A. Khlif, R. Abdelmalek, L. Ammari, B. Kilani, I. Boutiba-Ben Boubaker

**Affiliations:** 1 Laboratoire de résistance aux antimicrobiens LR99ES09, Université de Tunis El Manar, Faculté de médecine de Tunis, Tunisie; 2 Service de microbiologie, CHU Charles Nicolle, Tunis, Tunisie; 3 Service des maladies infectieuses, CHU La Rabta, Tunis, Tunisie

La résistance aux antirétroviraux (ARV) constitue un défi majeur dans la prise en charge des patients infectés par le VIH. Cette étude vise à décrire le profil des résistances du VIH-1 aux différentes classes d’ARV en Tunisie. Il s’agit d’une étude descriptive incluant 209 patients infectés par le VIH, réalisée sur une période de 12 mois (novembre 2023 à octobre 2024). Le génotypage a été réalisé à l’aide du kit HIV-1 *Genotyping with Integrase* (71,8%) ou en suivant le protocole de l’Institut de Santé Carlos III de Madrid (28,2%). L’analyse des résistances a été effectuée selon l’algorithme de Stanford (versions 9. 5. 1,9. 6 et 9. 8). Les mutations détectées ont été classées en trois niveaux de résistance: bas niveau (BNR), résistance intermédiaire (RI) et haut niveau de résistance (HNR). L’âge moyen des patients était de 35,5±11,4 ans avec un sex-ratio de 3,01. Le sous-type CRF02_AG était prédominant (69,4%), suivi des sous-types B (11,5%) et CRF06_cpx (7,6%). Une résistance à au moins une classe d’ARV a été observée chez 25,8% des patients dont 66,7% étaient naïfs de traitement. Les inhibiteurs non nucléosidiques de la transcriptase inverse (INNTI) étaient les plus touchés (22%), avec une résistance associée à la névirapine et à l’éfavirenz (10,5%) et un HNR à la névirapine (8,6%). La résistance aux inhibiteurs nucléosidiques de la transcriptase inverse a concerné 4,8% des cas avec un HNR principalement pour la lamivudine, l’emtricitabine, la didanosine et la stavudine (2,4% des cas chacun). Pour les inhibiteurs de la protéase, une résistance a été détectée chez 2,8% des patients et concernait le nelfinavir. Une multirésistance a été retrouvée chez 3,3% des patients, tous sous traitement. Un seul patient présentait une résistance à toutes les classes d’ARV. Cette étude révèle une prévalence significative des résistances aux ARV en Tunisie, particulièrement aux INNTI, avec une proportion notable chez les patients naïfs. Ces résultats renforcent l’importance du génotypage systématique avant l’initiation du traitement antirétroviral pour guider le choix thérapeutique.

# Research article: Prévalence de l’infection par le papiMomavirus humain chez les femmes dans la région de Ben Arous

**Published:** 2025-11-09

**Authors:** MY. Fekih, S. Lahmar, S. Dhraief, S. Belakhel, H. Bettaïeb, M. Khoudhaïer Tlili, L. Thabet

**Affiliations:** 1 Laboratoire de biologie médicale, Centre de traumatologie et des grands brûlés, Université Tunis el Manar, Faculté de médecine de Tunis, UR22SP03, Tunisie; 2 Service de gynécologie-obstétrique, Hôpital régional El Yasminet de Ben Arous, Tunisie; 3 Cabinet privé Dr Mouna Khoudhaier Tlili, Centre médical Maxula, Ben Arous, Tunisie

Le cancer du col de l’utérus est causé dans la majorité des cas par une infection à des génotypes de papillomavirus humain (HPV) à haut risque oncogène. Les nouvelles recommandations de la Haute autorité de santé française, instaurées en 2019, reposent sur la vaccination et sur la détection du génome viral par PCR en plus du frottis cervico-vaginal.

L’objectif de l’étude était d’estimer la prévalence et la distribution des génotypes du HPV en période pré-vaccinale chez des femmes dans la région de Ben Arous.

Nous avons mené une étude prospective, de juillet 2024 à février 2025, chez des patientes consentantes âgées de 30 à 65 ans, provenant des consultations externes du service de gynécologie de l’hôpital régional de Ben Arous et du cabinet d’un gynécologue privé. Pour chaque patiente, un écouvillonnage cervical a été réalisé et une fiche de renseignements a été remplie. La détection et le génotypage du HPV ont été faites par le kit HPV Direct Flow Chip de Vitro Master, Diagnostica permettant la détection et le génotypage simultané de 35 génotypes d’HPV: 18 HpV à haut risque oncogène et 17 HPV à bas risque oncogène. Au total, 86 tests ont été réalisés. Parmi ceuxci, 79 étaient validés. L’âge moyen des femmes étudiées était de 43,4 ans. La majorité avait un niveau socio-économique moyen (63,9%), pas d’antécédents pathologiques notables (93,5%) et jamais bénéficié d’un dépistage du cancer du col de l’utérus auparavant (81,8%). La prévalence globale du portage de l’HPV était de 16,5%. Les infections se répartissaient en 61,5% d’infections simples et 38,5% d’infections multiples (2 à 4 génotypes). Les tranches d’âges de 31 ans à 40 ans et de 41 ans à 50 ans étaient celles les plus représentées chez les patientes porteuses de l’HPV (38,5% chacune). La prévalence du portage de génotypes HPV à haut risque était de 11,3%. Le génotype à haut risque le plus fréquent était le génotype 51 avec une prévalence de 5% suivi des génotypes 16,26,35,39,53 et 66 retrouvés chacun chez 1,3% des patientes. Les HPV à faible risque étaient retrouvés chez 6,3% des patientes, dominés par les génotypes 44 et 55 (3,8%). La majorité des patientes porteuses de l’HPV avaient un col macroscopiquement sain (76,9%). Une exocervicite ou un polype accouché par le col étaient observés dans 7,7% des cas. Par ailleurs, nous avons observé une association statistiquement significative entre le portage de l’HPV et la notion de partenaires sexuels multiples. La prévalence de l’HPV était de 16,5% chez les femmes de la région de Ben Arous, avec une majorité de génotypes à haut risque. Cette étude doit être élargie à l’échelle nationale afin de définir les génotypes circulants et d’évaluer la couverture vaccinale, la vaccination étant programmée en avril 2025.

# Research article: Profil épidémiologique, clinique et microbiologique de la tuberculose humaine à *Mycobacterium bovis* dans la région de Sfax

**Published:** 2025-11-09

**Authors:** A. Znazen, L. Krichene, S. Maalej, A. Ghorbel, S. Smaoui, F. Messadi

**Affiliations:** 1 Laboratoire régional d’hygiène, CHU Hédi Chaker, Sfax, Tunisie; 2 Faculté de pharmacie de Monastir, Tunisie.

La tuberculose (TB) à *Mycobacterium bovis (Mb)* est une anthropozoonose préoccupante, transmise principalement par la consommation de produits laitiers non pasteurisés ou par contact avec du bétail infecté.

Le but de notre travail était de décrire le profil épidémiologique, clinique et microbiologique des cas de TB à *Mb* diagnostiqués dans la région de Sfax.

Il s’agit d’une étude rétrospective réalisée au Laboratoire régional d’hygiène de Sfax sur une période de 3 ans (2022-2024) et portant sur tous les prélèvements à culture positive à *Mb*. L’examen direct des échantillons a été effectué après coloration à l’auramine. Les cultures ont été réalisées sur milieux solides (Lowenstein-Jensen et Coletsos) et liquide [Mycobacterial Growth Indicator Tube (MGIT)]. L’identification a été réalisée sur la base des caractéristiques morphologiques, biochimiques et moléculaires. L’étude de la sensibilité des souches à l’isoniazide (INH), la rifampicine (RIF), la streptomycine, l’éthambutol et au pyrazinamide a été réalisée sur milieu MGIT. Toute résistance a été confirmée génotypiquement par la technique moléculaire Hain Genotype® MTBDR plus.

Sur un total de 417 cas de TB confirmée, 337 étaient dus à *Mb* (80,8%) et 80 cas (19,2%) étaient causés par *Mb*. Les formes extra-pulmonaires et pulmonaires des TB à *Mb* représentaient respectivement 75% (n=60) et 25% (n=20) des cas. Le sex-ratio était de 0,9. Parmi les formes extra-pulmonaires, la TB ganglionnaire était la plus fréquente (n=38; 48%), suivie par la TB ostéo-articulaire (n=9; 11%) et la TB intestinale (n=7; 9%). Les notions de consommation de produits laitiers non pasteurisés et de contact avec le bétail étaient rapportées par 24% et 14% des patients, respectivement. L’examen direct était positif dans 59% des cas (n=47). L’étude de sensibilité aux antituberculeux a révélé des taux de résistance à l’INH de 4% et à la RIF de 1%. Notre étude a révélé un taux important de TB pulmonaire et extra-pulmonaire due à *Mb* dans notre région, soulignant la nécessité pour les autorités sanitaires de mener des campagnes de sensibilisation sur les risques liés à la consommation de lait cru afin de réduire la transmission de cette espèce.

# Research article: Apport de la PCR en temps réel dans le diagnostic et le traitement de la tuberculose extra-pulmonaire au laboratoire de microbiologie du CHU la Rabta

**Published:** 2025-11-09

**Authors:** C. Jradi, O. Makhloufi, M. Amri, S. Abbes, M. Zribi

**Affiliations:** Laboratoire de microbiologie, CHU La Rabta, Tunis, Tunisie

Le diagnostic de la tuberculose extra-pulmonaire représente un défi majeur. La PCR en temps réel est un outil de diagnostic rapide de la tuberculose multirésistante, recommandé par l’OMS. L’objectif de cette étude est d’évaluer l’apport de la PCR en temps réel dans le diagnostic de la tuberculose extra-pulmonaire, en comparaison avec la culture bactérienne et la microscopie.

Au total, 221 prélèvements ont été collectés chez 215 patients suspectés de tuberculose extra-pulmonaire ou de résistance au traitement antituberculeux, d’août 2022 à décembre 2024. La détection moléculaire du complexe *Mycobacterium tuberculosis* et des mutations du gène rpoB associées à la résistance à la rifampicine a été réalisée à l’aide du test Xpert MTB/RIF Ultra. L’examen microscopique a été effectué par la coloration de Ziehl-Neelsen et la culture sur le milieu Lowenstein-Jensen.

L’âge moyen des patients était de 39 ± 18,4 ans, avec des extrêmes de 1 à 85 ans. Le sex-ratio (H/F) était de 0,65. Parmi les 213 patients, 43,2% provenaient du service des maladies infectieuses et 26,7% du service d’ORL. Les motifs de diagnostic les plus fréquents étaient la forme ganglionnaire (50,7%) et neuro-méningée (25,4%). Parmi les 112 patients avec des renseignements cliniques, 31 avaient des antécédents de tuberculose et 11 étaient immunodéprimés. En ce qui concerne la PCR, 127 prélèvements étaient négatifs, 74 positifs et 19 avaient des traces. L’examen direct réalisé sur 215 prélèvements était positif dans 11 cas (5%). La culture, réalisée dans 213 cas, était positive dans 12 cas (5,6%). La PCR en temps réel a permis de rattraper les faux négatifs par l’examen direct dans 61 cas et les faux négatifs par la culture dans 56 cas. Parmi les 59 cas dont la résistance à la rifampicine a été testée par la PCR, tous ont montré une sensibilité à ce médicament.

Pour la gestion de la tuberculose extra-pulmonaire, la PCR en temps réel permet un diagnostic rapide et une détection précoce des résistances à la rifampicine garantissant une prise en charge précoce et adaptée.

# Research article: Tuberculose ganglionnaire cervicale: facteurs influençant l’évolution

**Published:** 2025-11-09

**Authors:** M. Lajhouri, M. Selmi, A. Mediouni, H. Chahed, N. Beltaief

**Affiliations:** Service d’ORL et de chirurgie cervico-faciale, CHU La Rabta, Tunis, Tunisie.

La tuberculose ganglionnaire cervicale représente un véritable défi thérapeutique. En effet, en raison d’une réponse au traitement variable et d’une évolution souvent imprévisible, des ajustements du schéma thérapeutique standard, voire des chirurgies répétées, peuvent être nécessaires. L’objectif était de décrire les profils épidémiologiques, cliniques, thérapeutiques et évolutifs d>une série de cas de tuberculose ganglionnaire cervicale et d’évaluer les facteurs pouvant influencer la nature de l’évolution de la maladie lors d’une étude rétrospective et analytique menée au service d’ORL sur une période de 3 ans (2019-2021), incluant les patients traités pour une tuberculose ganglionnaire cervicale confirmée par un examen anatomopathologique.

Les patients étaient répartis en deux groupes selon la nature de l’évolution. Celle-ci était considérée comme favorable si la durée du traitement était inférieure à 9 mois sans recours à une chirurgie en cours de traitement. Elle était considérée comme défavorable si le traitement dépassait les 9 mois et/ou en cas de chirurgie supplémentaire. Une comparaison entre les deux groupes avec une étude des facteurs influençant l’évolution était réalisée.

La série étudiée comportait 102 patients (32 hommes et 70 femmes). La médiane d’âge était de 34,5 [extrêmes 8-83] ans. La majorité des patients (78%) n’avait pas d’antécédents médicaux particuliers ni d’infection connue par le VIH. La consommation de produits laitiers non pasteurisés était rapportée par 38,2% des patients. Les adénopathies prédominaient au secteur II, elles étaient souvent multiples (85%). Leur taille était supérieure à 3 cm dans 64% des cas. L’hypoéchogénicité (55%) et la nécrose (41%) étaient respectivement les aspects échographiques et scanographiques les plus fréquents. La culture bactériologique pratiquée dans 20,6% des cas était négative. Le geste diagnostique initial consistait en une adénectomie dans 68% des cas, un curage ganglionnaire dans 9% des cas et la mise à plat d’abcès froids avec un curetage biopsique dans 24% des cas. Une quadrithérapie antituberculeuse initiale était prescrite chez tous les patients. La prescription d’éthambutol était prolongée au-delà de 2 mois chez 64% des patients. L’évolution globale était jugée favorable chez 55% des patients. Les facteurs associés à une évolution défavorable étaient: l’aspect pathologique de la peau en regard des adénopathies, la nécrose ganglionnaire au scanner, la mise à plat d’un abcès froid, l’absence d’amélioration à 2 mois de traitement et la non-prolongation de l’éthambutol au-delà de 2 mois. Les facteurs associés à une évolution favorable étaient: la peau saine en regard des adénopathies, le curage ganglionnaire initial, l’évolution favorable à 2 mois de traitement et la prescription prolongée d’éthambutol.

Les résultats de notre étude soulignent d’une part le rôle crucial de la chirurgie dans la prise en charge de la tuberculose ganglionnaire cervicale et d’autre part l’importance de la prise en compte des particularités épidémiologiques de la tuberculose.
